# In Vivo Skin Hydrating Efficacy of Fish Collagen from Greenland Halibut as a High-Value Active Ingredient for Cosmetic Applications

**DOI:** 10.3390/md21020057

**Published:** 2023-01-17

**Authors:** Eva Martins, Rui L. Reis, Tiago H. Silva

**Affiliations:** 1 3B’s Research Group, I3Bs—Research Institute on Biomaterials, Biodegradables and Biomimetics, University of Minho, Headquarters of the European Institute of Excellence on Tissue Engineering and Regenerative Medicine, Avepark—Parque de Ciência e Tecnologia, Barco, 4805-017 Guimarães, Portugal; 2 ICVS/3B’s—PT Government Associate Laboratory, 4806-909 Braga/Guimarães, Portugal

**Keywords:** marine collagen, cytotoxicity, skin cosmetic formulation, hydrogel, fish by-products, in vivo assay, hydration

## Abstract

The industrial processing of fish for food purposes also generates a considerable number of by-products such as viscera, bones, scales, and skin. From a value-added perspective, fish by-products can act also as raw materials, especially because of their collagen content (particularly in fish skin). Interestingly, the potential of marine collagen for cosmetic applications is enormous and, remarkably, the extraction of this protein from fish skins has been established for different species. Using this approach, we investigated the integration of marine collagen (*COLRp_I*) extracted from the skin of the Greenland halibut as an active ingredient in a cosmetic hydrogel formulation. In this study, extracts of marine collagen at concentrations up to 10 mg/mL showed a non-cytotoxic effect when cultured with fibroblast cells for 3 days. In addition, marine collagen extract, when incorporated into a cosmetic hydrogel formulation, met criterion A of ISO 11930:2019 regarding the efficacy of the preservative system (challenge test). In addition, the cosmetic formulations based on marine collagen at dosages of 0.1, 0.25 and 0.5% were tested in a clinical study on the skin of the forearms of 23 healthy volunteers, showing a sightly hydration effect, suggesting its potential for beauty applications. Moreover, this work illustrates that the circular economy concept applied to the fish processing industry can represent important benefits, at innovation, environmental and economic levels.

## 1. Introduction

Collagen is an important structural protein that generally consists of three polypeptide chains connected by hydrogen bonds in a triple helix structure [1]. It is an abundant protein, accounting for up to 30% of all proteins in the human body. It is present in major connective tissues and provides strength and flexibility to tissues such as skin, tendons, cartilage, bones and ligaments [2,3]. Interestingly, the imbalance between collagen synthesis and degradation leads to various pathological phenomena in the major connective tissues [4]. Although 90% of collagen in the human body is type I collagen [5], 28 types of collagens have been described, which differ in structure, noting that they are collagen or other proteins containing collagen-like domains. Currently, collagen derived from cattle and pigs is used in medical care and industrial prospects, and its application is restricted by the risk of viral and prion infections, religious and ethical constraints, and customs [6]. Some manufacturers reject the use of mammal collagen and prefer other sources [4]. Therefore, marine collagen has emerged as an alternative collagen source [7] with fewer concerns about human infectious diseases such as bovine spongiform encephalopathy (BSE) transmission, transmissible spongiform encephalopathy (TSE), and foot-and-mouth disease (FMD) [8]. Collagen isolated from marine sources is used in the fields of food, bioengineering, pharmaceuticals, biomedical wound healing, and cosmetics [9,10,11]. Interestingly, marine collagen is also metabolically compatible, water soluble, lacks religious constraints and is considered free of animal pathogens [12,13,14]. In food sector, collagen is used as a gelatin precursor in the production of emulsions, foams, colloids, and biodegradable films [15] or as source of collagen peptides that can be absorbed into mucosa via a buccal delivery system [16].

In most cases, the production of collagen follows an acid-soluble or enzymatic procedure, and its isolation is well established mainly from skins of several fish species [17,18,19]. However, other extraction methods can be used to extract aquatic biopolymers such as ultrasonication [20], electrodialysis [21], CO_2_ supercritical fluid method [22]. In addition, the use of fishery and aquaculture by-products, including skin, scales, and bones is also a preferred choice, contributing to a more sustainable exploitation of the raw materials with environmental and economic benefits.

Marine collagen-based skin substitutes have been shown to lessen inflammation, encourage the formation of granulation tissue, and increase a healthy re-epithelialization with the proper development of skin appendages [23]. By imitating the natural skin structure, collagen-based skin replacements are a promising method of promoting wound healing [23]. Collagen gives skin its strength and stability and changes in this molecule play an integral role in the aging process [24]. In addition, collagen is a promising biopolymer for the removal of skin defects, deforming scars, pigmentation, as well as for the healing of wounds such as extensive burns, tendon surgery, and muscle injuries [25]. Remarkably, marine collagen exhibited identical properties to human collagen, which has great potential for developing a next-generation matrix for cell culture and regenerative medicine applications [26], Thus, it is a suitable candidate for hemostatic materials and applications in wound healing [27], while collagen-based gels play a role in the remodeling phase of the wound healing process by promoting extracellular matrix remodeling and accelerating wound closure of injured tissue [28,29]. In the cosmetic field, collagen can be used as an ingredient in the formulation of beauty products (creams, gels, masks, shampoos), with claimed action against wrinkling and aging, nourish, moisturize, retain moisture, and block ultraviolet (UV) rays [12,30,31], Moreover, it can increase the elasticity of the skin, make it soft and shiny, improve fibroblast production and consequently promote the renewal of the extracellular matrix of the skin. Collagen-rich extracts can also be used in hair products to increase the resistance of strands, which contributes to the growth and strength of hair, but also for other wellbeing uses, such as to strengthen nails [32,33]. The most effective cosmetic products are gels containing collagenous material (mostly collagen hydrolysis products), whose triple helix structure is maintained in the skin due to the positive transdermal effect. These products allow the control of degradation at the skin surface through the formation of peptide chains that can penetrate the epidermis. Currently, there are cosmetic products on the market that use collagen of marine origin in their composition, which is mainly isolated from the skin and scales of fish [4].

Greenland Halibut *Reinhardtius hippoglossoides* (Walbaum, 1793) is a widespread commercially valuable species, which may be found in large quantities in the northernmost regions of the Atlantic and Pacific oceans as well as in the Arctic seas [34]. This species is also important groundfish fishery in Atlantic Canada [35]. Moreover, the high volume of fishing experienced by this species generates a huge quantity of by-products, namely skins, that should be valorized. This valorization cannot be only the production of compounds out of it, but also finding a use for those compounds. Our previous study demonstrated that skins from *Reinhardtius hippoglossoides* fish are a stunning source of collagen [36] with wide and impressive biotechnological prospects in fields such as cosmetical, nutraceuticals, and biomedical.

Untapped “blue” resources for various uses are abundant in marine environments. Greenland halibut may be a new alternative to provide collagen for conventional cosmetic applications because skins from halibut species are a very abundant by-product in fisheries due to its high volume of fishing [37,38]. In this study, the collagen isolated from the skin of the Greenland halibut was used as a raw material for a cosmetic formulation. Our work intends to fully recycle wastes from the fish plant industry and reconvert them in high added value. We aimed to show that the produced marine collagen was a suitable ingredient for use in the formulation of beauty applications for skin care, assessing its processability as ingredient of a formulation for cosmetic application and further evaluating its efficacy on skin hydration.

## 2. Results and Discussion

### 2.1. Characterization of Collagen Extracted from Greenland Halibut Skins

**Figure 1:**
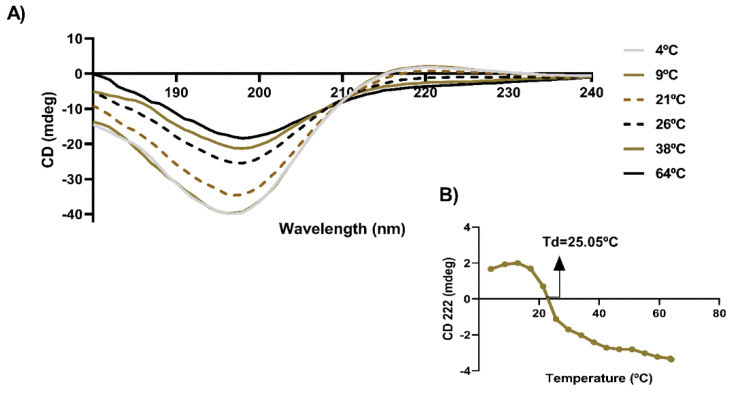
Effect of temperature on structural conformation of Greenland halibut collagen (*n* = 3). (**A**) Circular dichroism spectra obtained at different temperatures in the range 4–64 °C. (**B**) Variation of molar ellipticity at 222 nm with temperature for marine collagen and its relation with denaturation temperature.

Marine collagen was extracted from the skins of Greenland halibut and characterized envisaging its incorporation in a cosmetic gel formulation. Initially, the effect of temperature over protein structural organization was evaluated by measurements of circular dichroism (CD) molar ellipticity (θ) as a function of temperature between 4–64 °C. The CD spectra obtained at temperatures between 4–21 °C (Figure 1A) showed a weak positive absorption peak between 220–222 nm, indicating the presence of triple helix (i.e., collagen in its native form) and a negative one at 197 nm, associated with random coil conformation, with a crossing point (zero rotation) at 216 nm. The molar ellipticity at 222 nm decreased with the increment of temperature, being no longer positive at approximately 25.0 °C and above, indicating a breakdown of the triple helix structure (Figure 1B). The loss of triple helix corresponds to the denaturation of the protein and thus the transition temperature is associated to the denaturation temperature, herein determined as 25 °C. This value was determined using collagen solution diluted in acetic acid. We considered that the use of preservative materials in the hydrogel formulation will enable the incorporation of marine collagen as an active ingredient in low concentration for application in human body, envisaging their use in the cosmetic field.

**Figure 2:**
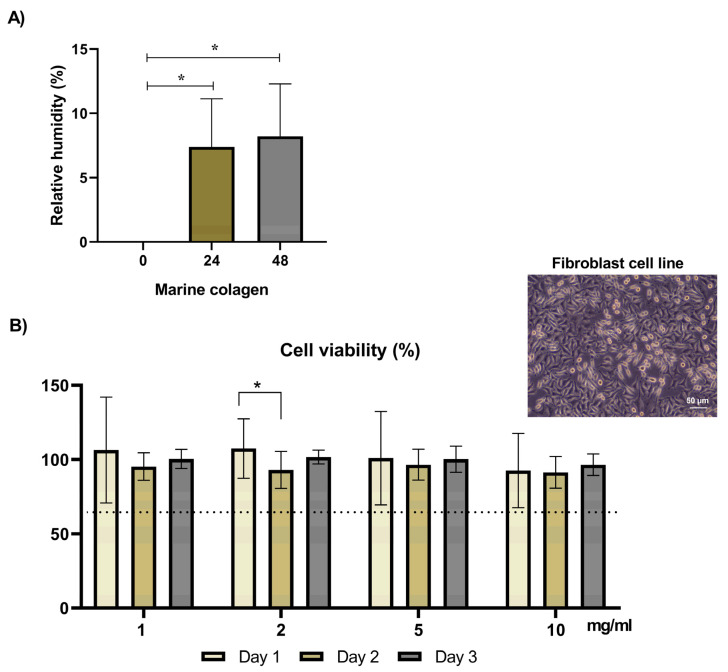
Collagen properties evaluation. (**A**) Moisture regains (%) of collagen under saturated atmosphere (*n* = 3). (**B**) Cell viability of fibroblast l929 cell line culture in the presence of collagen extracts at 1, 2, 5 and 10 mg/mL at different time points. Data are presented as mean ± standard error (*n* = 3), all samples contain statistical significance of * (*p* < 0.05).

The capacity of the produced collagen to retain moisture from the atmosphere under controlled humidity was evaluated during 48 h in a controlled humidity system maintained over a saturated CaCl_2_ solution. Collagen was capable to absorb moisture at about 8% of its weight, as shown by the statistically significant differences observed between the 0–24 h *(p* = 0.03) or 0–48 h (*p* = 0.013) (Figure 2A). This is an important indication considering collagen as potential ingredient for cosmetics regarding humectant applications—showing not only the potential to retain moisture but also to absorb water from the atmosphere—with the findings being in line with the values observed with codfish collagen [39]. Water is essential for maintaining the flexibility and integrity of skin. However, normal skin surface water content is low (10–15%) [40].

In addition, the eventual cytotoxic effect of collagen extracts over L929 fibroblast cells was evaluated by assessing the influence of the addition of collagen to the cell culture medium in the viability of the referred cells. The results of cell metabolic activity, determined by the MTS assay, after culturing the cells in the presence of varying concentrations of collagen extracts (Figure 2B) evidenced that collagen is not cytotoxic after 3 days of incubation, for all extracts concentrations tested (1, 2, 5 and 10 mg/mL), as the values of cell viability were always higher than 70% of the control (no collagen added). These results are compatible to the ones observed for other fish collagen extracts, namely from codfish (*Gadus morhua*) swim bladders [22], codfish [18] and small-spotted catshark (*Scyliorhinus canicular*) skins [41].

### 2.2. Marine Collagen as Ingredient of a Cosmetic Formulation

#### 2.2.1. Preliminary Stability Study

**Figure 3:**
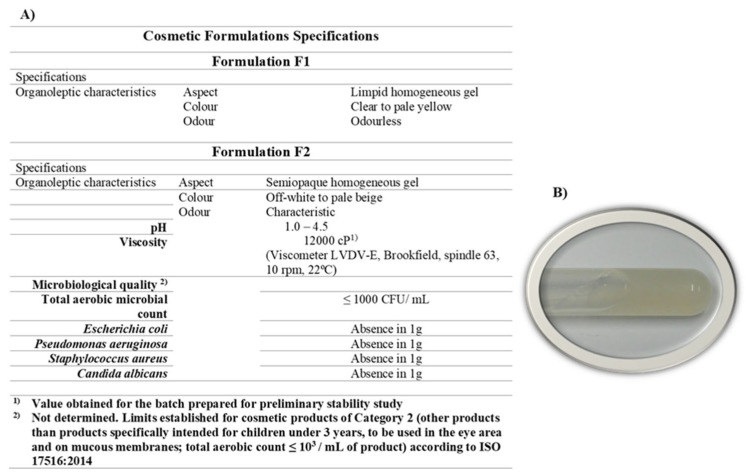
Formulation characteristics. (**A**) Organoleptic characteristics of formulation F1 (placebo) and F2 with marine collagen incorporated. (**B**) Image of the gel resulting from the incorporation of 10% (*w*/*w*) of the 5% marine collagen solution (F2).

For preliminary stability, two formulations were prepared: F1, a placebo formulation resulting in a clear viscous gel; and F2, a hydrogel formulation with marine collagen being incorporated at 0.5% *w*/*w* (F2), resulting in a homogeneous semi-opaque viscous gel (Figure 3). The use of a 5% marine collagen solution was revealed to be adequate to proceed with formulation development, enabling the incorporation at least up to 10% *w*/*w* into the final formulation.

**Figure 4:**
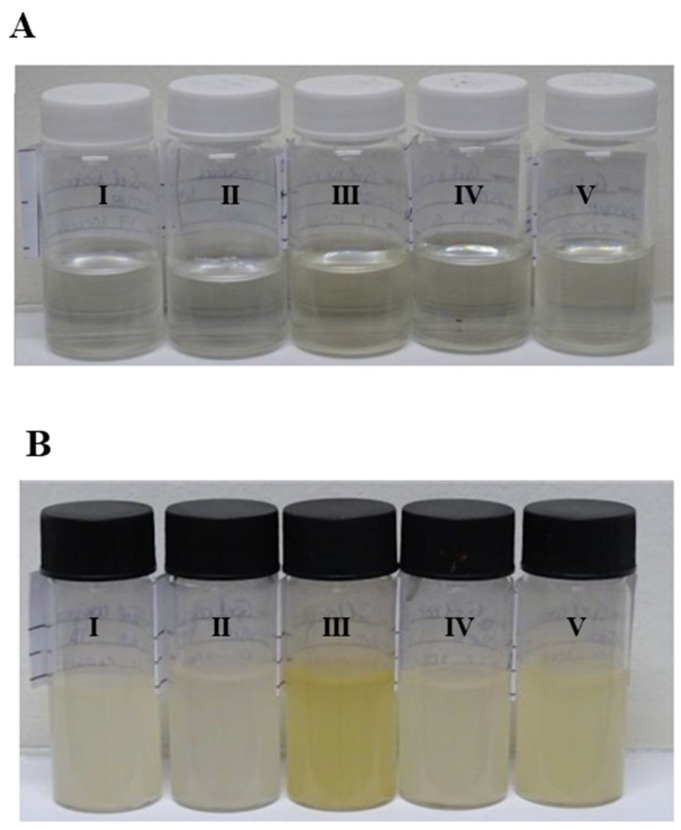
Organoleptic evaluation of the cosmetic formulations. (**A**) F1, placebo; and (**B**) F2—with 0.5% (*w*/*w*) marine collagen as an active ingredient, when stored, for one month: (I) at room temperature (22 ± 2 °C), (II) at refrigerated temperature (4 ± 2 °C), (III) at high temperature (40 ± 2 °C) or (IV) exposed to sunlight; and (V) when submitted to temperature cycles.

The initial pH of the base formulation F1 was neutral, around 7.3, but decreased with the incorporation of marine collagen to around 4.3. The pH range of 4.0–4.5 is compatible with the normal skin pH, and as described in the literature, skin with pH values below 5.0 is beneficial regarding barrier function, moisturization, and scaling. Furthermore, an acid skin pH such as 4.0–4.5 is more skin microbiome-friendly since it keeps the resident bacterial flora attached to the skin [42]. As a control, formulation F1 (placebo) was subjected to a preliminary stability investigation, with Table 1 summarizing the main findings of the study. F1 was resistant to stress, both mechanical and thermal induced by centrifugation and temperature cycles, respectively, and remained stable after a month of storage under various conditions. When compared to the initial hue, there was a small yellowing in the product stored at high temperatures, but it was still deemed acceptable (Figure 4A). The stress conditions (centrifuge and temperature cycles) were also resisted by formulation F2 (containing marine collagen as an active ingredient), although a slight yellowing occurred. Even when held at refrigerated temperature, room temperature, and exposed to sunlight for one month, it retains its organoleptic features. After 1 month of storage, organoleptic characteristics remain unaltered for the product stored at refrigerated temperature, room temperature, and exposed to sunlight, even though a slight yellowing occurred. The odor of the product became slightly more intense at all conditions except at refrigerated temperature, which is not considered relevant. pH values also remained within the pH range specified for all conditions tested. The color of the product stored at high temperature was highly altered (more yellow) and considered not stable at 40 °C (Figure 4B). This result is predictive of its stability in the long term, and it is recommended to store the formulation with active ingredients at refrigerated temperature. Nevertheless, it is advisable to perform additional stability studies to evaluate the stability profile of the active ingredient individually and/or in solution/dispersion. If lower concentrations of marine collagen in the formulation were studied, a less intensity of yellowing would be expected.

#### 2.2.2. Preservation Efficacy Test According to ISO 11930

**Figure 5:**
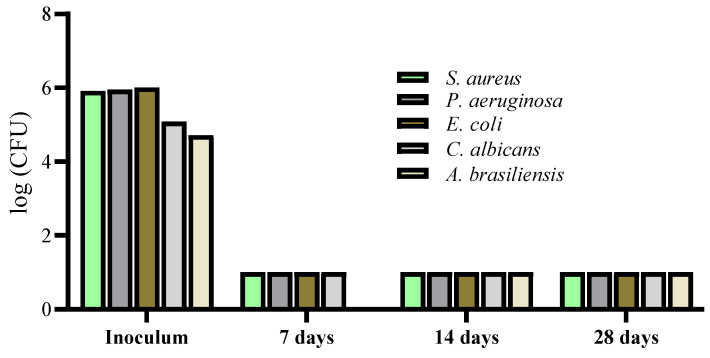
Challenge test and microorganism’s evaluation on the formulation F2. Chart representing the variation of CFU log values of the five microorganisms tested.

Formulation F2 was then submitted to the preservation efficacy test (challenge test) to assure its microbiological safety, before proceeding to clinical study for the evaluation of the skin hydrating potential. The results of the challenge test are depicted in Figure 5. Moreover, the results showed the evolution of a colony-forming unit of the five microorganisms included in the challenge test, from the initial inoculum until 28 days, assessed on the formulation F2 (Figure 5).

According to the ISO 11930 regarding the evaluation of the antimicrobial protection of a cosmetic product, the formulation F2 complies with criterion A for five microorganisms. It has a protective effect against microbial proliferation, namely *Staphylococcus aureus*, *Pseudomonas aeruginosa*, *Escherichia coli*, *Candida albicans*, and *Aspergillus brasiliensis* that may present a potential risk for the user. For *S. aureus*, *P. aeruginosa*, *E. coli*, *C. albicans* and *A. brasiliensis*, the product complies a log reduction of 4.92 (≥3), 4.95 (≥3), 5.01 (≥3), 4.08 (≥1) 3.72 (≥0) respectively after 7 days and no increase after 14 and 28 days (Figure 5). *S. aureus* and *P. aeruginosa* are considered to be part of the “typical” resident skin flora, however, the most prevalent bacterial genera found on human skin is *Staphylococcus* [43,44]. *S. aureus* is typically considered of as a pathogen, however since more methicillin-resistant strains are discovered inhabiting the moist skin regions of healthy individuals [45]. The microbiological quality of F2 was confirmed, being determined as within the limits established for cosmetics products of Category 2 (other than products specifically intended for children under 3 years, to be used in the eye area and on mucous membranes; total aerobic count ≤ 10^3^/mL of product) according to the ISO 17516:2014 (Cosmetics—Microbiology—Microbiological limits, ISO/TC 217 Cosmetics, 2014).

### 2.3. Evaluation of the Hydrating Efficacy of Skin Cosmetic Formulation Comprising Marine Collagen

The clinical study evaluated the hydrating efficacy of the produced marine collagen incorporated in a finished gel cosmetic formulation at three different concentrations: 0.5%, 0.25% and 0.1% *w*/*w* (formulations designated as F2, F3 and F4). Forearms of twenty-three healthy volunteers were randomized selected for the measurements of the hydration levels after applications of the six products evaluated in this study. Measurements of the skin capacitance were performed comparing the test formulations to two negative controls (placebo and type II water) using a corneometer that determine the quantitative assessment of the average hydration level of the skin surface (stratum corneum) in the volunteers [46]. The stratum corneum, which serves as the skin’s barrier, is made up of a cornified layer of dead cells with high protein content (corneocytes) that are embedded in a lipid matrix [47]. The measurements of skin hydration enable us to determine the slight differences among the tested products.

**Figure 6:**
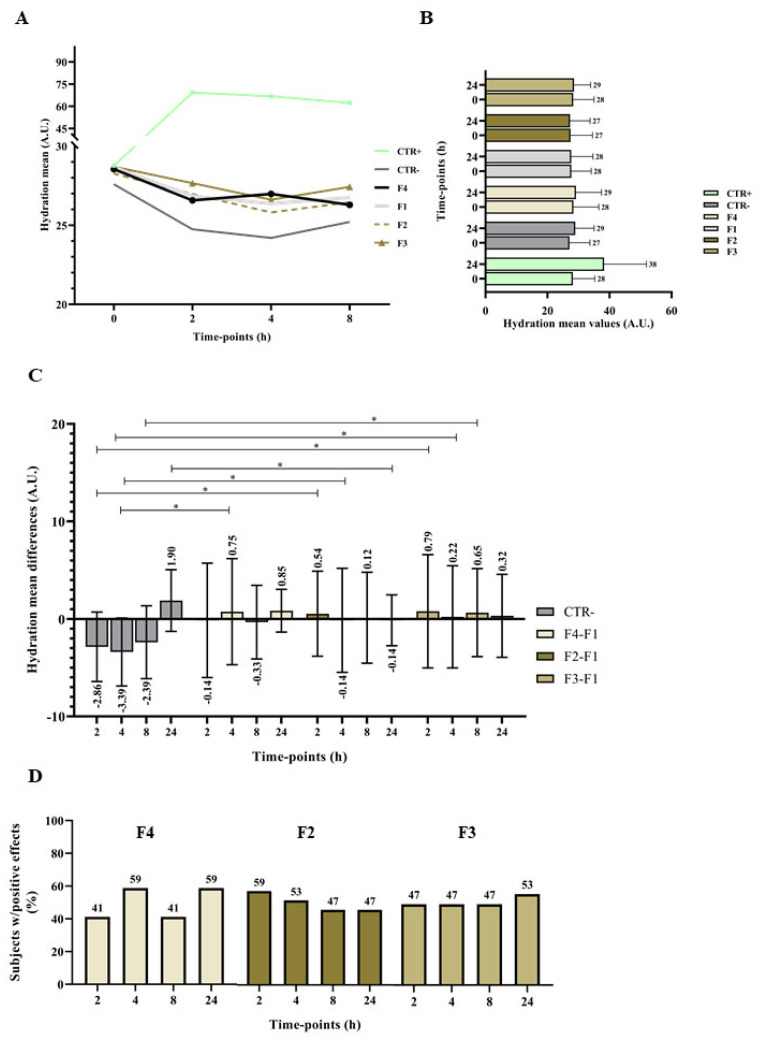
Skin hydration means results obtained before and after 2, 4, 8 h (**A**), and 24 h (**B**) of products administration. Skin hydrations mean differences to the baseline obtained before and after 2, 4, 8, and 24 of treatment negative control (code: B), the deduction of the placebo (**D**) results to the Gel F4 results, to the Gel F2 results and the Gel F3 results (**C**). Data are presented as mean ± standard error (*n* = 3), all samples contain statistical significance of * (*p* < 0.05). Results of the effect of marine collagen enriched formulation on skin hydration, subtracting the placebo for each time point, indicated as the percentage of subjects with positive effects (**D**).

To guarantee that all test sites were in the same hydration conditions before the study beginning a statistical comparison of the hydration mean values was performed. For the time-point t0* a *p*-value of 0.996 in a Kruskal–Wallis Test was achieved and for the time-point t0** a *p*-value of 0.993 was obtained in an ANOVA showing all test skin sites were in the same conditions in the study beginning. The results obtained at all time points for the skin hydration with each product/control are presented in Figure 6. The mean differences presented were determined by subtracting time-point 0 (before products’ application) results to the results obtained 2, 4, 8, and 24 h after the administration of the products or subtracting the placebo effect to the values of the investigational products at all time-points.

The stratum corneum of the skin’s surface layer, which may be measured for moisture, contains vital details on the biophysical characteristics and operation of the skin barrier [48]. It is important for cosmetic products that the active ingredients remain in the skin, penetrate sufficiently deeply without going too deep and causing systemic availability [49]. A statistically significant difference was found between the positive control and the negative control at all time points evaluated, confirming the adequacy of the methodology used in the study. The hydration values obtained for the placebo showed some fluctuations throughout the study period. Therefore, to properly evaluate the effect of marine collagen enrichment of the cosmetic formulations on skin hydration, the differences attributed to the placebo were subtracted from the differences determined after the administration of the product. Slight improvements in hydration were obtained when using the formulations comprising marine collagen (Figure 6C), revealing the potential of collagen as ingredients for use in cosmetic purposes. Although statistically significant differences were only observed for some time points, namely: F2 promoted an increase in hydration by 0.54 (*p*-value = 0.049) after 2 h; F3 promoted an increase in hydration by 0.79 (*p*-value = 0.046), 0.22 (*p*-value = 0.021) and 0.65 (*p*-value = 0.028) after 2, 4 and 8 h, respectively; and F4 promoted an increase in hydration by 0.75 (*p*-value = 0.030) after 4 h. These findings suggest that marine collagen has a moisturising effect on the skin, with a long-lasting (up to 8 h) effect, particularly when marine collagen is present in the formulation at 0.25%, but no dose-dependent correlation was detected, within the range of concentrations tested and under the conditions of this study.

## 3. Materials and Methods

### 3.1. Marine Collagen Extraction

**Figure 7:**
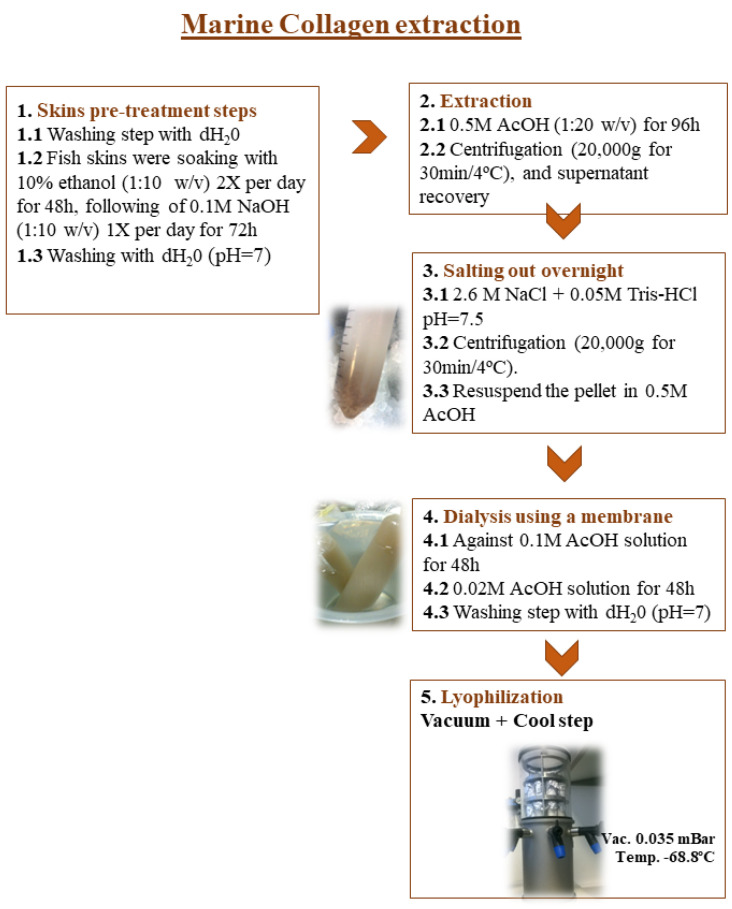
Experimental steps for the extraction of marine collagen from the skins of Greenland halibut.

The skins of the Greenland halibut (*Reinhardtius hippoglossoides*) were used as raw material for the extraction of collagen. Experimental extraction was carried out as previously described in Martins et al. 2022 [36] and illustrated in the scheme in Figure 7. All operations were performed at 4 °C to avoid denaturation of collagen.

Skins were washed with distilled water and immersed in 10% ethanol (1:10 *w*/*v*) for fat removal (24 h), 0.1 M NaOH (1:10 *w*/*v*) solution for 72 h to remove non-collagenous proteins, and cold distilled water until neutral pH. The collagen was then extracted with 0.5 M acetic acid (1:20 *w*/*v*) (96 h) and centrifugated at 20,000× *g* for 30 min at 4 °C. The supernatant was salted out with 2.6 M NaCl + 0.05 M Tris-HCl pH = 7.5, and the pellet was recovered after centrifugation, at 20,000× *g* for 30 min at 4 °C and resuspended in 0.5 M acetic acid solution. The collagen solution was dialyzed against 0.1 M acetic acid for 2 days, 0.02 M acetic acid for 2 days, and then against water. The samples were stored at −80 °C and lyophilized. After lyophilization, collagen samples were stored at room temperature until further analysis.

### 3.2. Collagen Characterization

Thermal behavior by circular dichroism, moisture regain, and cytotoxicity were evaluated to determine the properties of marine collagen prior to incorporation into a cosmetic formulation.

#### 3.2.1. Circular Dichroism

The collagen solution was placed in a quartz cell (2 mm) and the CD spectra were measured using a spectrometer (J-1500, JASCO, Tokyo, Japan). The lyophilized collagen was dissolved in 50 mM acetic acid at a concentration of 0.1 mg/mL. The CD spectra were recorded between 180 and 240 nm, at defined temperatures within the range 4–64 °C, with a scanning speed of 50 nm/min. The denaturation temperature (Td) of collagen was determined at a fixed wavelength of 222 nm.

#### 3.2.2. Moisture Uptake Capacity

Moisture absorption capacity was evaluated by the change in weight after incubation of the freeze-dried collagen in a controlled humidity atmospheric system. Prior to testing, lyophilized collagens (triplicates) were dried for 72 h in the oven at 30 °C and then pre-weighted (T0). The samples were transferred to a closed system with 60% constant relative humidity and sustained calcium chloride at room temperature. After 24 and 48 h, respectively, the samples were reweighed, and the moisture absorption capacity was calculated using the following equation and expressed as a percentage of the dry weight:

$$\mathrm{Moisture}\;\mathrm{absorption}\;\mathrm{capacity}\;\left(\%\right)=\frac{\;\mathrm{weight}\;\mathrm{of}\;\mathrm{collagen}\;\mathrm{upon}\;\mathrm{incubation}-\mathrm{dry}\;\mathrm{weight}\;\mathrm{of}\;\mathrm{collagen}}{\mathrm{dry}\;\mathrm{weight}\;\mathrm{of}\;\mathrm{collagen}}\times 100$$

#### 3.2.3. In Vitro Assay of the Cytotoxicity of Collagen Extracts

The fibroblast cell line (L929 cells) purchased from the European Collection of Cell Cultures (ECACC, UK) was grown in Dulbecco’s Modified Eagle Medium (D- MEM low glucose, Sigma-Aldrich, St. Louis, MO, USA) supplemented with 10% fetal bovine serum (FBS, Biochrom, Cambridge, UK) and 1% penicillin-streptomycin (Life Technologies, Carlsbad, CA, USA), pH 7.4, at 37 °C in a humidified atmosphere with 5% CO_2_ until cells reached 90% confluence. Cells were used in passages 38–40 and harvested at 25,000 cells per well in 24-well plates. The cells were allowed to adhere for 24 h, and then collagen extracts were added to the adherent cells. The collagen extracts were dissolved in the culture medium at concentrations of 1, 2, 5, and 10 mg/mL for 24 h. For all tests, a control was performed without the presence of collagen extracts in the culture medium. Each experimental condition was tested in triplicate and three independent assays were performed. The viability of cells cultured with different concentrations of collagen extracts was determined after 1, 2 and 3 days using the MTS assay (CellTiter 96^®^ Aqueous One Solution Cell Proliferation Assay, Promega, Madison, WI, USA). At each time point, the culture medium was removed, and the cells were rinsed with sterile phosphate-buffered saline. A mixture of culture medium (without FBS and phenol red) and MTS reagent (5:1 ratio) was added to each well and left to react for 4 h, at 37 °C, in a humidified 5% CO_2_ atmosphere. The absorbance in blanks (MTS medium only), controls and samples were read in triplicate at 490 nm in a microplate reader (Synergy HT, Bio-TEK, Winooski, VT, USA), to assess the quantity of purple formazan product formed, directly proportional to the number of metabolically active/living cells in culture. Cytotoxicity is considered when cell viability is below 70% of the control.

### 3.3. Marine Collagen Evaluation for a Cosmetic Purpose

**Figure 8:**
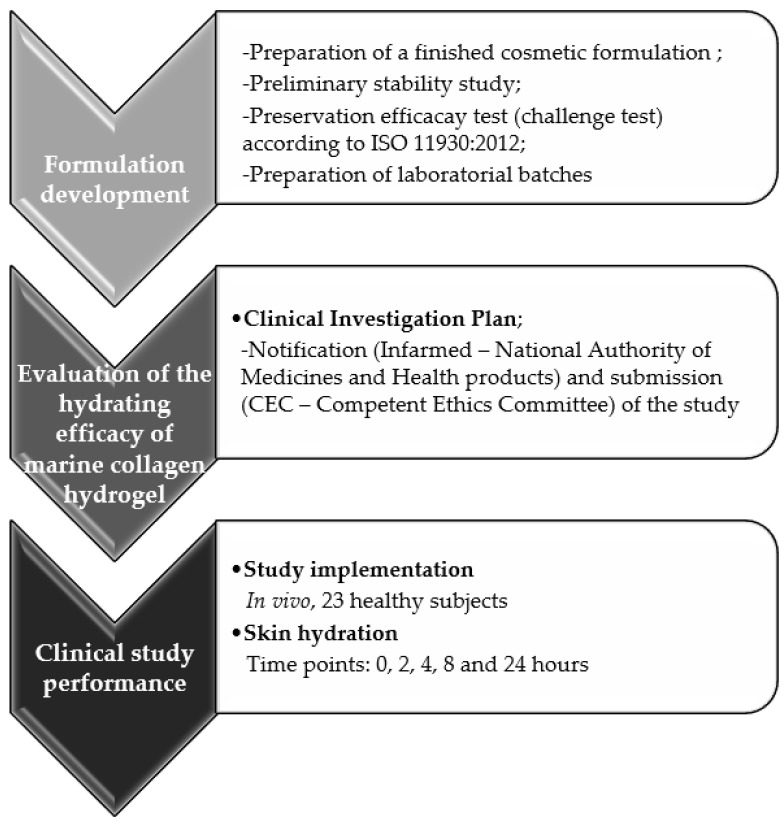
The methodology for evaluating the cosmetic potential of marine collagen as an active ingredient in a hydrogel on the skin of healthy individuals.

The produced collagen (*COLRp_I*) was tested as an active ingredient for incorporation into a finished cosmetic formulation to evaluate its preliminary stability and preservative activity for use in a blinded, controlled, randomized clinical study in accordance with Regulation (EC) No. 1223/2009. The study was performed to evaluate the moisturizing effect of the collagen hydrogel-based cosmetic product after one single application for 24 h (Figure 8).

#### 3.3.1. Incorporation of Marine Collagen on a Cosmetic Formulation

Marine collagen was incorporated into a cosmetic base emulsion formulation. The formulation was developed and optimized considering the dissolution, compatibility and stability properties of the marine collagen, while minimizing the addition of ingredients with hydrating effects to demonstrate the active effect of the collagen used. The hydrogel was prepared using water as solvent, gelling agent and preservative system. For this purpose, hydroxyethylcellulose (HEC) was used as a nonionic gelling agent, which is also commonly used in cosmetic or pharmaceutical formulations [50,51] and is relatively stable at pH 2–12 without affecting the viscosity of the solutions. The viscosity of aqueous HEC solutions decreases with increasing temperature, in a reversible process in which the original viscosity is restored after cooling. Aqueous solutions of HEC should be preserved for prolonged storage and are compatible with a variety of water-soluble antimicrobial preservatives [52]. Sodium benzoate and potassium sorbate, a synergistic combination widely used in cosmetics, pharmaceuticals, and foods, were used as the preservative system. The water-soluble preservatives do not affect the surface tension [53,54]. Both the HEC and preservative systems are non-toxic and non-irritating at the recommended concentrations for use on the skin. They are used according to Regulation (EC) No. 1223/2009.

Two cosmetic formulations were prepared, namely:

(a) **Formulation F1**—base formulation or placebo, consisting of type II water HEC (Natrosol™ 250M from Ashland, supplied by Safic Alcan Portugal, Milheirós, Portugal) at 2.0%, sodium benzoate at 0.42%, and potassium sorbate at 0.21% dissolved in type II water. For preliminary stability study, pH was adjusted to approximately pH 4 with citric acid in solution (0.8 mL to 100 g);
(b) **Formulation F2**—based on base formulation (F1) but with 10% *w*/*w* of the pre-dispersion of collagen (5% *w*/*v* in acetic acid 0.5 M), thus corresponding to a formulation with 0.5% *w*/*w* of marine collagen. The concentration of collagen tested in the formulation was selected after an extensive literature review of cosmetic products available on the market Table S1 (Supplementary SI).

#### 3.3.2. Preliminary Stability Studies

In the case of a cosmetic formulation, the preliminary stability was evaluated with respect to physical stress, temperature cycles and storage of the manufactured formulations (F1 and F2). The formulations were subjected to the following stress conditions to evaluate the preliminary stability; Physical stress (centrifugation)—the samples were visually analyzed at each 30-min cycle of the 10 centrifugation cycles (at 3000 rpm, for a total of 5 h); Temperature cycling—the samples were subjected to physicochemical analysis (organoleptic properties and pH) before and after 8 cycles from 24 to 72 h at 4 °C and 40 °C, respectively; Storage at room temperature (22 ± 2 °C), refrigerated temperature (4 ± 2 °C), high temperature (40 ± 2 °C) and direct sunlight for one month—the samples were subjected to physicochemical analysis (organoleptic properties and pH) before and after one month of storage under each condition. The formulation that best withstands the above stress conditions was selected for the evaluation of the preservation efficacy test.

#### 3.3.3. Preservation Efficacy Test (challenge Test)

The efficacy of the preservative system of the formulation F2 was tested according to the ISO 11930—Evaluation of the antimicrobial protection of a cosmetic product and the acceptance criteria (Table 2). This is a mandatory test for cosmetic products marketed in the European Union, to ensure that they can preserve possible microbiological contamination of the cosmetic product in accordance with the regulation (EC) No. 1223/2009. The preservative properties of the preparation are adequate if, under the test conditions, the number of microorganisms in the inoculated preparation significantly decreases or does not increase after the times prescribed in the selected reference standard. For each study, the log reduction for each microorganism was determined at each time point and compared to the acceptance criteria. The test product was inoculated separately with each of the test microorganisms at a ratio of 200 μL of calibrated cell suspension per 20 g of test product (Pour Plate Technique). Products meeting criterion A show a better log reduction profile over time, products meeting only criterion B still show an acceptable log reduction when other microbiological risk control factors are associated with the product.

Before starting the efficacy test, the mesophilic aerobic bacteria according to ISO 21149 and the yeasts and molds according to ISO 16212 were counted in duplicate by placing them in a suitable culture medium (Sabouraud dextrose agar, or Potato dextrose agar, or Tryptic soy agar (Prolabo, France)). The total aerobic plate count is equal to the sum of the bacterial, yeast and mold plate counts. The microorganisms tested and incubation conditions tested were for *Staphylococcus aureus* ATCC 6538—32.5 ± 2.5 °C, 2 days; *Pseudomonas aeruginosa* ATCC 9027—32.5 ± 2.5 °C, 2 days; *Escherichia coli* ATCC 8739—32.5 ± 2.5 °C, 2 days; *Candida albicans* ATCC 10231—32.5 ± 2.5 °C, 2 days and *Aspergillus brasiliensis* ATCC 16404—22.5 ± 2.5 °C, 5 days.

For the validation of assay conditions, the test product was diluted in neutralizing agent (buffered peptone water containing 3% polysorbate 80, 0.3% soy lecithin, 3% saponins and Triton X100, Prolabo, France) at 0.1%; and inoculated individually with the known concentration of the test microorganisms. The recovery capacity of each of the tested microorganisms in the product was evaluated after placing an aliquot in the appropriate culture medium and compared to the recovery capacity of these microorganisms in the neutralizer in the absence of the test product.

#### 3.3.4. Clinical Study for the Evaluation of the Hydrating Efficacy of a Cosmetic Ingredient

A single blinded study was performed to evaluate the in vivo skin hydration effect. Twenty-three healthy individuals (women and men) with a mean age of 41 ± 12.6 years participated in the study, in accordance with the Declaration of Helsinki. Informed consent was obtained from all volunteers. They were asked to not apply any topical products in the forearms 24 h before the beginning and throughout the test period. The study was conducted to evaluate hydrating efficacy of six products in vivo, as listed in Table 3, corresponding to:

(I) an active ingredient (marine collagen) incorporated in a finished cosmetic formulation at three concentrations (0.10%, 0.25%, and 0.50%), obtained by the addition of a 5% *w*/*v* marine collagen solution, previously prepared in 0.5 M acetic acid, at 2.0%, 5.0%, and 10.0% *w*/*w*, respectively.
(II) a cosmetic placebo formulation, corresponding to the same finished cosmetic formulation without the active ingredient and used as negative control;
(III) deionized water (type II), used as another negative control;
(IV) aqueous solution of glycerin at 85%, used as positive control.

**Table 3:** Products for cosmetic evaluation in clinical Investigation Plan.

Investigational and Comparator Products	Product Codification
Cosmetic placebo hydrogel	F1
Cosmetic hydrogel with 0.50% marine collagen	F2
Cosmetic hydrogel with 0.25% marine collagen	F3
Cosmetic hydrogel with 0.10% marine collagen	F4
85% glycerine aqueous solution as positive control	CTR+
Type II water as negative control	CTR-

##### Mode of Application—Test Area

**Figure 9:**
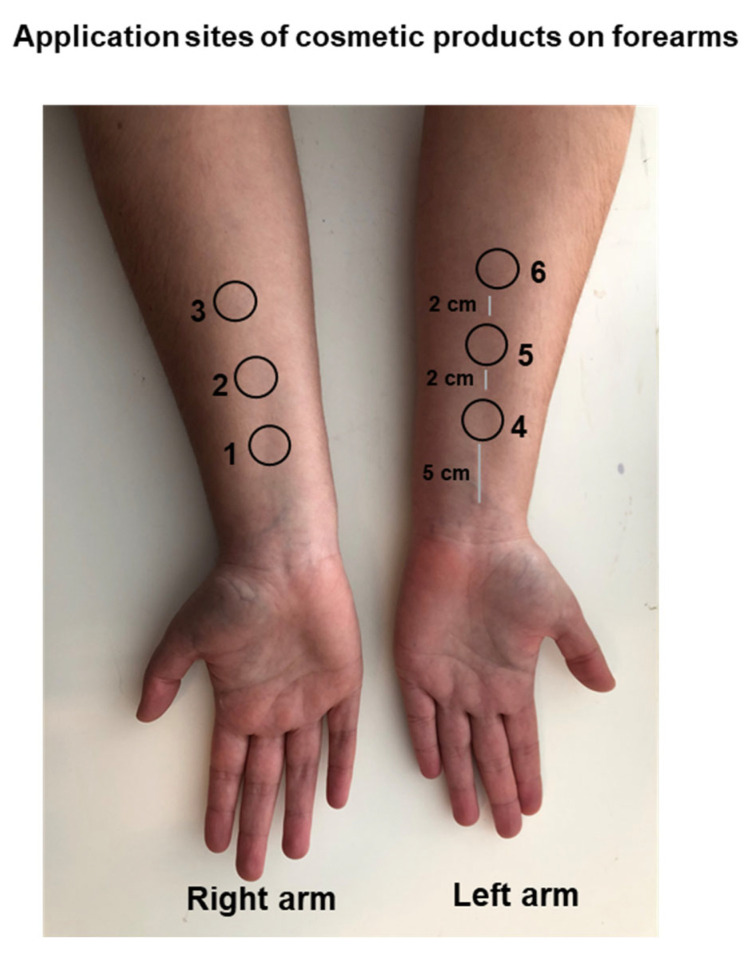
Skin sites on the forearms for the application of six (1, 2, 3, 4, 5 and 6) products.

The forearms of the subjects that meet the criteria for participation in the study (see Supplementary SII) were marked with six test areas for application (Figure 9) and rinse-off products were applied to damp skin at 2 μL/cm^2^ (approximately 14 mg of products), let on for a few minutes and then rinsed off with water. For a randomized trial, the respective product code was noted near the test area. The forearm was carefully wiped with absorbent paper before applying the products.

##### In Vivo Skin Hydration

The subjects were acclimatized to the room conditions for 20 min before the measurements. Skin hydration was investigated using a Corneometer^®^ CM825 (Courage+Khazaka electronic GmbH, Köln, Germany) coupled to a multiple probe for skin measurements. Capacitance changes, depending almost solely upon the water content in the stratum corneum, are detected and evaluated. Measurements were applied slight pressure in each test site (triplicates), before application of the products (T0) and at 2, 4, 8, and 24 h. Corneometer^®^ is based on a capacitive measurement principle. Different capacitance changes are converted into a digital measured value (arbitrary units), which is proportional to the skin humidity. The range of variation of the values of skin hydration degree is between 0–120 arbitrary units (AU) and were measured in a controlled temperature (23.0 ± 1.0 °C) and relative humidity (50.0 ± 10.0%).

### 3.4. Statistical Analysis

Statistical analysis was performed using GraphPad Prism 8 software. All the performed tests were conducted in triplicate (n = 3). Regarding the descriptive statistical analysis of the skin, hydration results were performed at each time-point of evaluation. Normality tests (Shapiro–Wilk test) were performed to assure normal distribution of the data obtained. If normal distribution of the data was verified, Student’s t-tests were conducted to compare: (i) the skin hydration values obtained with the negative control (type II water) versus with positive control, at each time-point of evaluation, to validate the methodology; (ii) the skin hydration values obtained with the investigational products versus with each negative control (placebo and type II water), at each time-point of evaluation, to prove the hydrating efficacy of each investigational product, individually; For non-normal distributions non-parametric tests (Wilcoxon test) was used. The significance value was established at 0.05 and a power of 0.95.

## 4. Conclusions

The efficiency of the methodology previously established for the isolation of collagen from the skin of the Greenland halibut, *R. hippoglossoides*, was confirmed, obtaining a product with denaturation temperature of 25 °C, as determined by CD, revealing no cytotoxicity and capable to absorb atmospheric moisture up to a value equivalent to 50% of its own weight. These features suggested its potential use as an active ingredient for the development of a skin cosmetic formulation (hydrogel) with humectant effect The additions of a preservative system in the cosmetic formulation enable to the improvement of the stability of the final product and its use at human body temperature. In this regard, an established skin cosmetic gel was enriched with marine collagen at 0.5% *w*/*w*, thus proposing a new formulation fairly resistant to mechanic and thermal stress and capable to be stored during at least one month, at room temperature or chilled environments. Moreover, a single administration of the developed marine collagen-enriched formulation enhanced skin hydration, with the effect being felt until up to 8 h, although the outcome did not reveal a dose-dependent performance. Overall, the obtained results highlight the importance of the skin of the Greenland halibut under the concept of circular economy, with the study herein reported disclosing a methodology to add value to a material currently regarded as by-product from fish processing industry, by using it as raw material for the sustainable production of collagen with applicability as bioactive ingredient in cosmetics and skincare formulations. Our findings may be of significant interest to biotech and processing companies that turn waste materials into high-quality, creative innovative products for cosmetic purposes. Furthermore, collagen ingredient is demonstrated to be safe, low cost, and stable in formulations. We considered that the methodology presented for the extraction of Halibut collagen in this study could be applied to different common commercial species caught and used for human consumption which generates a high amount of byproducts.

## Figures and Tables

**Table 1:** Preliminary stability results of the hydrogel formulations F1 (placebo) and F2 (with marine collagen).

					Time Zero		Temperature Cycles				1 Month							
	Parameters		Methods	Specifications	Room Temperature (22 ± 2 °C)		Room Temperature (22 ± 2 °C)		End of the 8th Cycle		Room Temperature (22 ± 2 °C)		Refrigerated Temperature (4 ± 2 °C)		High Temperature (40 ± 2 °C)		Sunlight Exposure	
											Mean	SD	Mean	SD	Mean	SD	Mean	SD
**Hydrogel Formulation F1 (Placebo)**	**Organoleptic characteristic of the product**	**Aspect**	Organoleptic evaluation	Limpid homogeneous gel	Conform		Conform		Conform		Conform		Conform		Conform		Conform	
		**Colour**		Clear to pale yellow	Conform		Conform		Conform		Conform		Conform		Conform		Conform	
		**Odour**		Odourless	Conform		Conform		Conform		Conform		Conform		Conform		Conform	
	**pH**	**T = 22 ± 1 °C**	Procedure PE 01 (pH 1000 L phenomenal VWR)	-	3.86	0.01	4.00	0.00	3.99	0.00	4.00	0.00	4.03	0.00	3.00	0.00	3.97	0.00
					-		-		-		-		-		-		-	
	**Physical stress**	-	10 cycles of centrifuge at 3000 rpm, during 5 h (Centrifuge Compact Star CS4, VWR)	Without a change in the appearance	Conform		-											
**Hydrogel Formulation F2 (with marine collagen)**	**Organoleptic characteristic of the product**	**Aspect**	Organoleptic evaluation	Semi-opaque homogeneous gel	Conform		Conform		Conform		Conform		Conform		Conform		Conform	
		**Colour**		Off-white to pale beige	Conform		Conform		Conform (slightly more yellow)		Conform (slightly more yellow)		Conform		Non conform (more yellow)		Conform (slightly more yellow)	
		**Odour**		Characteristic	Conform		Conform		Conform (very slightly more intense)		Conform (very slightly more intense)		Conform		Conform (slightly more intense)		Conform (very slightly more intense)	
	**pH**	**T = 22 ± 1 °C**	Procedure PE 01 (pH 1000 L phenomenal VWR)	4.0–4.5	4.27	0.00	4.41	0.00	4.41	0.01	4.41	0.00	4.45	0.01	4.39	0.00	4.40	0.00
					Conform		Conform		Conform		Conform		Conform		Conform		Conform	
	**Physical stress**	-	10 cycles of centrifuge at 3000 rpm, during 5 h (Centrifuge Compact Star CS4, VWR)	Without changes in the appearance	Conform		-											

**Table 2:** Acceptance criteria of the preservation efficacy test according to ISO 11930:2019.

Log Reduction Values (R_x_ = lg_N0_-lg_NX_) Required ^a^								
Microorganisms	Bacteria			*C. albicans*			*A. brasiliensis*	
Sampling Time	T7	T14	T28	T7	T14	T28	T14	T28
Criterion A	≥3	≥3 And NI ^b^	≥3 And NI	≥1	≥1 And NI	≥1 And NI	≥0 ^c^	≥1
Criterion B	-	≥3	≥3 And NI	-	≥1	≥1 And NI	≥0	≥0 And NI

^a^ In this test, an acceptable range of deviation of 0.5 log is accepted; ^b^ NI: no increase in the count from the previous contact time; ^c^ R_x_ = 0 when lg_N0_ = Ig_Nx_ (no increase from the initial count).

## Supplementary Materials

The following supporting information can be downloaded at: https://www.mdpi.com/article/10.3390/md21020057/s1. Supplementary data can be consulted in Supplementary SI—“Cosmetic product information”, SII—“Subjection selection”, SIII—“Products weight analysis”, SIV—“Temperature and relative humidity in vivo assay” and SV—“Skin hydration results”. Table S1: Organoleptic characteristics and technical description of cosmetic products available on the market which have marine collagen ingredients.
