# Supplementary material for: In Vivo Skin Hydrating Efficacy of Fish Collagen from Greenland Halibut as a High-Value Active Ingredient for Cosmetic Applications
Source: Mar Drugs. 2023 Jan 17;21(2):57. doi: 10.3390/md21020057 (PMC9960085; doi:10.3390/md21020057)
Supplement: Supplementary file 1 [file marinedrugs-21-00057-s001.zip › marinedrugs-2147075-supplementary.pdf]

Supplementary Materials

Supplementary Material SI – Cosmetic product information

SI – Technical information of marine-derived cosmetic product

Table S1. Organoleptic characteristics and technical description of cosmetic products available on the market which have marine collagen ingredients.

Commercial name	INCI name	Producer	Use level (%)	Soluble collagen content (%)	Organoleptic characteristics
**AC Marine Collagen PF**	Soluble collagen (98%), leuconostoc/radish root ferment filtrate	Active concepts	1.0 - 10.0	1.0	Hazy viscous liquid
**Pancogene marin 2**	Aqua, soluble collagen, phenoxyethanol	Gatefosse	2.0 - 5.0	0.3	Opalescent liquid
**Collagen CLR (extracted from calf skin in a weakly acidic buffer without using enzymes)**	Soluble collagen sodium benzoate	CLR Chemisches Laboratorium Dr. Kurt Richter GmbH	3.0 – 10.0	0.5	Transparent, colourless to yellowish, at room temperature slightly viscous liquid
**Collasol™ M PE**	Aqua, soluble collagen	Croda	-	0.5	-
**Maricol S (Tuna)**	Aqua, soluble collagen, sodium benzoate	Croda	-	0.1 – 1.0	Water-clear, almost transparent, viscous liquid, almost odourless

**Ichtyocollagene**	Aqua, propyleneglycol, soluble collagen	Sederma	2.0 – 6.0	0.4 – 0.7	Clear to slightly opalescent pale yellow liquid
**Bio-marine collagen 6PH**	Soluble collagen	Cobiosa	0.5 – 5.0	5.0 – 7.0	-
**Collaplex 1.0**	Aqua, soluble collagen, phenoxyethanol, methylparaben, ethylparaben	Gfn-Selco	-	1 – 1.3	Opaque, viscous solution
**Collaplex 1.0 PHE**	Aqua, soluble collagen, phenoxyethanol	Gfn-Selco	1.0 – 5.0	-	-
**Marine collagen**	Aqua, soluble collagen	I.R.A. Istituto Ricerche Applicate	0.5 – 5.0	3.0	-

Supplementary Material SII - Subject selection

S2 - Subjects' information

The subjects were selected according to following inclusion and exclusion criteria.

Inclusion criteria for Group I: Gender: Female/Male; Age: 18 years old and over; Having signed an Informed Consent Form (ICF); Willingness, ability and likeliness to comply with all the study procedures and restrictions; Available for the entire study duration; Understanding Portuguese language: Portuguese-speaking subjects capable of reading the documents;

Exclusion criteria for Group I: Known allergy or known history of hypersensitivity to the components of the investigational products, comparative products, controls and/or related compounds; Currently participating in another clinical study that may interfere with the study; having diseases that may impair the study; Under pharmacological treatments that may impair the study; - Having applied any type of topical product on the test region (forearms) within the 24 h preceding the first performance study visit; Diagnosed skin diseases and/or cutaneous alterations on the test region (forearms) that can impair the study (E.g. scars, tattoos, marks, burns, excessive body hair); Pregnancy or intention to conceive during the study; Breast-feeding.

Subjects’ general health data (including diseases and frequent medication), consumer habits and concomitant medications are recorded on the Case Report Form (CRF).

The following instructions and advice were given to the included subjects:

- Subjects must not apply any topical product (cosmetic, pharmaceutical or otherwise) in the evaluation area (forearms), since the 24 hours that precede the first performance study visit (including cosmetic products such as hydrating products, body lotions, shower gels, soaps, etc) and until the end of the study;
- On the first performance study visit, subjects will remain for approximately 9 hours in a temperature and humidity-controlled room; during this time, food and drinks will be provided to all subjects according to their needs;
- In case of adverse events, subjects should inform immediately the research centre;
- Subjects must inform the Investigators in case of any change in their health status or medication;
- Female subjects must inform the Investigators if they become or are found to be pregnant during participation in the study;
- Male subjects must inform the Investigators if their partner becomes or is found to be pregnant during the subject's participation in the study;
- Subjects must address to the study facilities at the scheduled date and time;
- Subjects should be careful in washing their hands as to avoid splashes of water with detergents/soaps/gels during the study performance;
- Subjects must not wash their forearms during the study performance;
- Subjects should keep the forearms exposed (not covered with clothes) and avoid touching with the volar part of the forearm in any surface during the first 9 hours of the study;

- During the study performance, subjects must not have bath and shall avoid the direct contact of water and cleansing products with the forearms;

S2.1 - Subject´age

Table S2.1 – Information regarding the subject´ age

Subject #	Subject ID	Subjects' Age on recruitment day
1	1	41
2	2	58
3	4	30
4	5	40
5	6	32
6	9	49
7	12	55
8	13	64
9	14	44
10	15	36
11	16	40
12	18	37
13	19	19
14	20	60
15	21	51
16	23	29
17	24	61
18	25	27
19	26	32
20	27	23
21	28	40
22	29	45
23	30	33

**Mean ± SD**	41±12.6

S2.2 - Diseases

Table S2.2 – Information regarding the subject´ diseases

Subject ID	Diseases	
	Do you have any diagnosed disease?	If yes, specify:
1	No	
2	No	
4	No	
5	No	
6	No	
9	No	
12	No	
13	No	
14	Yes	Asthma; Hypertension; Allergic rhinitis
15	No	
16	Yes	Bipolar disorder
18	No	
19	No	

20	No	
21	No	
23	Yes	Hypertension
24	No	
25	No	
26	No	
27	No	
28	Yes	Chronic ulcerative colitis
29	Yes	Chronic hives
30	No	

S2.3 - Allergies

Table S2.3 – Information regarding the subject´ allergies

Subject ID	Allergies			
	Do you have any diagnosed allergy?	If yes, specify:	Have you ever had any allergy or intolerance to [cosmetic products	If yes, specify:

			or medicines]?	
1	No		No	
2	No		No	
4	No		No	
5	No		No	
6	No		No	
9	Yes	Rhodium	No	
12	No		No	
13	No		No	
14	No		No	
15	No		No	
16	No		No	
18	No		No	
19	Yes	Fleas	No	
20	No		No	
21	Yes	Milk	No	
23	Yes	Penicillin	No	
24	No		No	
25	Yes	Metals, sweat	No	

26	Yes	Pets' hair, Pollen	Yes	Product with retinoids
27	No		No	
28	No		No	
29	Yes	Dust	No	
30	No		No	

S2.4 – Exposure to sunlight, UV light and pollution

Table S2.4 – Information regarding the subject´ exposure to sunlight, UV light and pollution

Subject ID	Exposure to sunlight, UV light and pollution		
	Daily sunlight exposure:	Frequency of solariums:	Frequency of locals with high levels of pollution (eg.: a lot of smoke or dust):
1	Rare	Null	Rare
2	Rare	Null	Rare
4	Rare	Null	Rare
5	Rare	Null	Rare
6	Rare	Null	Rare
9	Sporadic	Null	Rare

12	Rare	Null	Rare
13	Rare	Null	Rare
14	Rare	Null	Rare
15	Rare	Null	Rare
16	Rare	Null	Sporadic
18	Rare	Null	Rare
19	Rare	Null	Rare
20	Rare	Null	Rare
21	Rare	Null	Rare
23	Sporadic	Null	Rare
24	Sporadic	Null	Rare
25	Rare	Null	Rare
26	Rare	Null	Rare
27	Rare	Null	Null
28	Rare	Null	Rare
29	Sporadic	Null	Null
30	Rare	Null	Rare

S2.5 – Consumption patterns

Table S2.5 – Information regarding the subject´ consumption patterns

Subject ID	Consumption patterns				
	Smoke habits:	If yes, approximately, for how long (years)	If yes, approximately how many cigarettes do you smoke per day	Alcohol intake:	Coffee intake:
1	No			Once to twice a month	Never
2	No			Frequent	Daily
4	No			Very rarely or never	Daily
5	No			Very rarely or never	Never
6	No			Once to twice a week	Never
9	No			Very rarely or never	Daily
12	No			Once to twice a week	Daily
13	No			Once to twice a week	Daily
14	No			Very rarely or never	Never
15	No			Very rarely or never	Daily
16	Yes	20 years	5 to 10 days	Once to twice a week	Daily
18	No			Once to twice a month	Never
19	No			Once to twice a month	Never
20	No			Very rarely or never	Never

21	No			Once to twice a week	Daily
23	No			Once to twice a month	Daily
24	No			Once to twice a month	Daily
25	Yes	10 years	<5 days	Once to twice a week	Daily
26	No			Once to twice a month	Daily
27	No			Once to twice a month	Daily
28	No			Once to twice a week	Daily
29	No			Very rarely or never	Never
30	No			Once to twice a month	Never

S2.6 – Concomitant Medication

Table S2.6 – Information regarding the subject´ concomitant medication

Subject ID	CM No.	Medication name (Record 'specify Generic or Brand' name)	Start Date	Stop Date	Ongoing at the end of the study?	Reason for use (Enter related AE diagnosis, or other reasons for use, e.g. Prophylaxis)	Dose (units)	Route	Frequency
14	1	Pulmicort	NK/NK/2017	-	Yes	Asthma	1	Oral	2x/day
14	2	Bisoprolol	NK/09/2018	-	Yes	Hypertension	1	Oral	1x/day
15	1	Minigeste	NK/09/2011	-	Yes	Contraceptive	1	Oral	1x/day
16	1	Diplexil	NK/NK/1999	-	Yes	Bipolar disorder	1	Oral	1x/day
16	2	Seroquel	NK/09/2009	-	Yes	Bipolar disorder	1	Oral	1x/day
16	3	Triminulet	NK/NK/2010	-	Yes	Contraceptive	1	Oral	1x/day
18	1	Gynera	NK/NK/2002	-	Yes	Contraceptive	1	Oral	1x/day
23	1	Indapamide 1.5 mg	NK/02/2019	-	Yes	Hypertension	1	Oral	1x/day
23	1	Azalia	NK/11/2018	-	Yes	Contraceptiv	1	Oral	1x/day

						e			
25	1	Minigeste	NK/NK/2009	-	Yes	Contraceptive	1	Oral	1x/day
26	1	Benzoyl Peroxide	15/10/2019	-	Yes	Acne	1	Topical	1x/day
28	1	Budenofalk	NK/NK/2016	-	Yes	Chronic ulcerative colitis	1	Rectal	Every two days
29	1	Yaz	01/10/2017	-	Yes	Contraceptive	1	Oral	1x/day
29	2	Atarax	01/02/2019	-	Yes	Rhinitis	1	Oral	1x/day
29	3	Bisoprolol 2.5 mg	15/09/2019	-	Yes	Tachycardia	1	Oral	1x/day
30	1	Daylette	NK/NK/2010	-	Yes	Contraceptive	1	Oral	1x/day

Supplementary Material SIII – Products weight analysis S3.1 - Investigational Products Weight

Table S3.1 – Information regarding the F4 weight

F4		
Subject ID	Product No.	Weight at t0 (mg)
1	1	13.9

2	1	13.9
4	1	14.2
5	1	13.9
6	1	14.0
9	1	14.1
12	1	14.0
13	1	14.0
14	1	13.9
15	1	13.9
16	1	14.1
18	1	14.0
19	1	14.0
20	1	14.1
21	1	14.1
23	1	13.9
24	1	14.0
25	1	13.9
26	1	14.0
27	1	13.9
28	1	14.1
29	1	13.9
30	1	13.9

Table S3.2 – Information regarding the F1 weight

Product F1		
**Subject ID**	**Product No.**	**Weight at t0 (mg)**
1	1	13.9
2	1	14.0
4	1	13.9
5	1	14.0
6	1	14.0
9	1	14.0
12	1	13.9
13	1	14.1
14	1	14.0
15	1	14.1
16	1	14.1

18	1	13.9
19	1	13.9
20	1	13.9
21	1	14.1
23	1	13.9
24	1	13.9
25	1	14.1
26	1	14.0
27	1	14.1
28	1	13.9
29	1	13.9
30	1	13.9

Table S3.3 – Information regarding the F2 weight

F2		
**Subject ID**	**Product No.**	**Weight at t0 (mg)**
1	1	14.1
2	1	13.9
4	1	14.0
5	1	13.9
6	1	14.1
9	1	13.9
12	1	14.1
13	1	13.9
14	1	13.9
15	1	14.1
16	1	13.9
18	1	13.9

19	1	13.9
20	1	14.1
21	1	13.9
23	1	13.9
24	1	14.0
25	1	13.9
26	1	14.1
27	1	14.1
28	1	14.0
29	1	14.1
30	1	14.0

Table S3.4 – Information regarding the F3 weight

F3		
**Subject ID**	**Product No.**	**Weight at t0 (mg)**
1	1	14.0
2	1	14.1
4	1	14.1
5	1	14.1
6	1	13.9
9	1	14.1
12	1	13.9
13	1	13.9
14	1	14.1
15	1	13.9

16	1	14.0
18	1	13.9
19	1	13.9
20	1	14.1
21	1	14.0
23	1	14.0
24	1	14.1
25	1	14.1
26	1	14.1
27	1	14.0
28	1	14.1
29	1	14.0
30	1	13.9

Supplementary Material SIV – Temperature and relative humidity in vivo assay S4.1 – Temperature and relative humidity

Table S4.1 – Information regarding the temperature (ºC)

Subject ID	T0*	T0**	T24
1	22.0	-	23.9***
2	23.7	23.7	23.5

4	22.0	.	24.0***
5	23.3	23.3	23.6
6	22.5	-	23.9***
9	24.0	24.0	23.7
12	22.4	-	23.8***
13	23.7	23.7	23.7
14	22.2	22.2	24.9***
15	23.4	23.4	23.6
16	23.7	23.7	23.9
18	23.1	23.1	23.3
19	22.3	-	24.0***
20	24.0	24.0	23.8
21	22.9	22.9	23.5
23	23.9	23.9	23.9
24	23.5	23.5	23.6
25	-	24.0	24.6
26	-	24.0	24.8
27	-	24.0	24.5
28	-	24.0	24.3
29	-	23.9	24.0
30	-	24.0	24.3
*1st group values considered for the hydration evaluation of time-points 2h, 4h and 8h after products´application<br>** 2nd group: values considered for the hydration evaluation at time-point 24h after products´application<br>***Values not considered for the mean values			

Table S4.2 – Information regarding the relative humidity (%)

Subject ID	T0*	T0**	T24
1	59.8	-	45.9***
2	46.8	46.8	47.3
4	60.0	-	45.4***
5	50.3	50.3	44.3
6	59.9	-	47.4***
9	45.4	45.4	46.8
12	59.9	-	48.4***
13	46.8	46.8	47.4
14	60.0	-	45.4***
15	49.3	49.3	47.3
16	46.8	46.8	47.4
18	50.3	50.3	44.2
19	59.9	-	47.4***
20	45.4	45.4	46.9
21	52.3	52.3	46.3
23	46.9	46.9	47.4
24	46.4	46.4	46.3
25	-	59.9	60.9
26	-	60.0	61.1
27	-	59.1	61.0
28	-	59.1	62.0
29	-	60.0	59.2
30	-	60.0	60.9

*1st group values considered for the hydration evaluation of time-points 2h, 4h and 8h after products´application
** 2nd group: values considered for the hydration evaluation at time-point 24h after products´application
***Values not considered for the mean values

Table S4.3 – Information regarding the raw data temperature (ºC)

Subject ID	T0*	T0**	T2	T4	T8	T24
1	22.7	-	23.3	23.7	23.7	23.9***
2	23.9	23.9	23.3	23.1	23.2	23.8
4	23.5	-	23.6	23.6	23.4	23.4***
5	23.7	23.7	23.8	23.6	23.6	23.5
6	23.8	-	23.8	23.8	23.0	23.8***
9	23.6	23.6	23.7	23.2	23.8	23.9
12	24.0	-	23.9	23.8	23.3	23.7***
13	24.0	24.0	23.3	23.3	23.6	23.6
14	23.8	-	23.6	23.8	23.4	23.8***
15	23.6	23.6	24.0	23.7	23.4	23.9
16	23.9	23.9	23.3	23.3	23.8	24.0
18	23.3	23.3	23.7	23.5	23.3	23.6
19	23.9	-	23.7	24.0	23.0	24.0***
20	23.7	23.7	23.8	23.4	23.8	24.0
21	23.6	23.6	23.8	23.7	23.8	23.5
23	23.7	23.7	23.6	23.4	23.9	24.0
24	24.0	24.0	23.6	23.4	23.8	23.8
25	-	24.0	-	-	-	24.5
26	-	23.6	-	-	-	25.3
27	-	24.0	-	-	-	24.5

28	-	23.4	-	-	-	25.0
29	-	23.6	-	-	-	25.3
30	-	24.0	-	-	-	24.6
*1st group values considered for the hydration evaluation of time-points 2h, 4h and 8h after products´application<br>** 2nd group: values considered for the hydration evaluation at time-point 24h after products´application<br>***Values not considered for the mean values						

Table S4.4 – Information regarding the raw data relative humidity (%)

Subject ID	T0*	T0**	T2	T4	T8	T24
1	60.0	-	59.9	60.0	56.9	46.9***
2	46.4	46.4	46.8	50.8	50.3	46.3
4	59.0	.	59.3	59.9	56.3	46.8***
5	46.8	46.8	51.4	47.3	48.4	45.8
6	58.7	.	60.0	60.0	56.2	47.4***
9	50.4	50.4	48.9	48.8	48.9	46.9
12	58.7	-	58.7	60.0	56.7	47.9***
13	46.9	46.9	47.3	50.3	49.4	46.8
14	58.1	-	59.3	59.6	56.3	46.3***
15	46.3	46.3	48.4	49.4	48.3	47.4
16	46.4	46.4	47.3	50.3	48.4	47.4
18	50.3	50.3	52.4	50.9	48.3	43.8
19	58.2	-	59.1	59.5	57.3	47.4***
20	50.4	50.4	47.9	48.3	48.4	47.4
21	46.3	46.3	51.9	47.9	47.4	46.8
23	51.4	51.4	47.3	48.3	48.4	47.4
24	46.4	46.4	44.3	49.3	48.4	46.3

25	-	60.0	-	-	-	64.0
26	-	57.3	-	-	-	60.0
27	-	59.1	-	-	-	63.0
28	-	60.0	-	-	-	62.0
29	-	60.0	-	-	-	57.5
30	-	58.4	-	-	-	64.0
*1st group values considered for the hydration evaluation of time-points 2h, 4h and 8h after products´application<br>** 2nd group: values considered for the hydration evaluation at time-point 24h after products´application<br>***Values not considered for the mean values						

Table S4.5. Mean temperature and relative humidity values recorded during the acclimatization period

	**T0***		**T00****		**T24**	
	T (ºC)	RH (%)	T (ºC)	RH (%)	T (ºC)	RH (%)
Mean	23.1	52.1	23.6	52.0	23.9	51.6
SD	0.7	6.2	0.5	6.1	0.4	7.1
* 1sr group: values considered for the hydration evaluation at time-points 2, 4 and 8 h after product applications<br>* 2nd group: values considered for the hydration evaluation at time-point 24 h after products' application						

Table S4.6. Mean temperature and relative humidity values recorded during the measurements

	T0*		T00**		T2*		T4*		T8*		T24**	
	T (ºC)	RH (%)	T (ºC)	RH (%)	T (ºC)	RH (%)	T (ºC)	RH (%)	T (ºC)	RH (%)	T (ºC)	RH (%)
Mean	23.7	51.8	23.7	51.9	23.6	52.4	23.5	53.0	23.5	51.4	24.2	51.9
SD	0.3	5.6	0.2	5.8	0.2	5.7	0.2	5.3	0.3	4.0	0.6	7.7
* 1sr group: values considered for the hydration evaluation at time-points 2, 4 and 8 h after product' applica<br>* 2nd group: values considered for the hydration evaluation at time-point 24 h after products' application												

Supplementary Material SV – Skin hydration results

S5.1 – Hydration values

Table S4.1 – Information regarding the hydration (A.U.) values from all the subjects at time zero (T0), and .after application of the product A, 2h, 4h, 8h and 24h.

CTR+					
Subject	t0	t2	t4	t8	t24
**1**	27.60	77.10	76.00	75.80	24.40
**1**	24.50	76.00	76.20	76.00	18.30
**1**	35.00	76.00	76.30	77.30	27.70
**2**	32.60	81.50	77.90	84.40	44.00

**2**	36.00	76.90	78.70	80.60	48.20
**2**	35.00	77.00	85.80	79.80	51.80
**4**	37.70	76.40	77.70	75.50	39.80
**4**	38.70	76.10	75.80	76.40	42.00
**4**	36.90	76.10	76.00	77.40	39.60
**5**	18.60	78.10	77.60	37.10	33.50
**5**	21.60	77.30	77.30	38.30	30.10
**5**	23.30	77.40	78.80	40.10	34.10
**6**	27.70	76.10	76.40	89.80	33.00
**6**	22.90	76.30	76.30	85.60	34.40
**6**	23.60	76.30	76.50	73.60	41.00
**9**	33.90	77.00	61.80	75.40	32.90
**9**	34.10	69.20	72.70	77.10	36.60
**9**	31.30	72.50	69.00	79.40	35.70
**12**	34.70	76.10	76.40	82.30	41.20
**12**	36.80	76.10	76.30	77.40	39.50
**12**	37.30	76.10	76.30	78.20	43.50
**13**	26.70	40.90	37.10	41.30	34.90
**13**	26.80	40.00	36.30	41.80	30.20
**13**	28.20	41.10	40.90	41.80	34.30
**14**	28.30	76.40	69.50	75.10	29.70
**14**	31.30	80.50	74.90	73.20	35.40
**14**	29.20	76.30	73.20	69.30	30.70

**15**	15.40	29.50	25.70	26.40	17.90
**15**	16.80	36.20	30.50	29.50	22.20
**15**	13.80	31.50	29.50	26.60	21.00
**16**	31.70	76.90	89.60	69.00	44.60
**16**	33.00	77.60	75.20	71.90	40.90
**16**	32.90	84.30	73.50	76.40	39.50
**18**	27.90	76.80	74.40	37.60	32.50
**18**	26.80	76.90	77.60	37.20	33.30
**18**	28.40	77.00	77.10	45.80	36.40

CTR+					
**Subject**	**t0**	**t2**	**t4**	**t8**	**t24**
**19**	28.70	76.40	76.10	74.90	31.20
**19**	30.30	76.20	77.00	72.60	33.10
**19**	30.10	76.40	76.40	68.80	30.70
**20**	28.80	68.40	74.20	90.30	31.20
**20**	31.10	77.10	78.00	85.20	36.90
**20**	31.30	77.10	77.40	83.40	40.00
**21**	28.00	76.60	39.90	33.80	32.30
**21**	25.00	65.20	41.30	32.40	33.60

**21**	26.00	76.70	43.20	38.10	36.00
**23**	18.60	37.60	27.90	30.30	25.50
**23**	15.50	34.00	23.40	30.30	26.00
**23**	17.80	37.90	24.50	32.70	29.60
**24**	37.10	77.10	88.30	71.10	39.40
**24**	34.70	77.10	97.40	63.20	41.70
**24**	36.60	77.00	79.20	68.50	43.60
**25**	38.70	-	-	-	35.60
**25**	39.30	-	-	-	34.90
**25**	40.30	-	-	-	36.90
**26**	30.80	-	-	-	84.00
**26**	28.70	-	-	-	83.20
**26**	32.40	-	-	-	82.20
**27**	19.00	-	-	-	26.30
**27**	17.30	-	-	-	26.50
**27**	18.50	-	-	-	27.80
**28**	23.10	-	-	-	34.10
**28**	27.50	-	-	-	37.60
**28**	26.60	-	-	-	39.80
**29**	29.10	-	-	-	33.70
**29**	25.80	-	-	-	33.10
**29**	24.50	-	-	-	36.10
**30**	35.90	-	-	-	46.20

**30**	35.50	-	-	-	49.30
**30**	38.80	-	-	-	53.80

Table S5.2 – Data analysis AADE of the hydration values from all the subjects at time zero (T0), and .after application of the CTR+, 2h, 4h, 8h and 24h.

CTR+						
**Subject**	**t0***	**t0****	**t2***	**t4***	**t8***	**t24****
**1**	27.60	-	77.10	76.00	75.80	-
**1**	24.50	-	76.00	76.20	76.00	-
**1**	35.00	-	76.00	76.30	77.30	-
**2**	32.60	32.60	81.50	77.90	84.40	44.00
**2**	36.00	36.00	76.90	78.70	80.60	48.20
**2**	35.00	35.00	77.00	85.80	79.80	51.80
**4**	37.70	-	76.40	77.70	75.50	-
**4**	38.70	-	76.10	75.80	76.40	-
**4**	36.90	-	76.10	76.00	77.40	-
**5**	18.60	18.60	78.10	77.60	37.10	33.50
**5**	21.60	21.60	77.30	77.30	38.30	30.10
**5**	23.30	23.30	77.40	78.80	40.10	34.10
**6**	27.70	-	76.10	76.40	89.80	-
**6**	22.90	-	76.30	76.30	85.60	-
**6**	23.60	-	76.30	76.50	73.60	-

**9**	33.90	33.90	77.00	61.80	75.40	32.90
**9**	34.10	34.10	69.20	72.70	77.10	36.60
**9**	31.30	31.30	72.50	69.00	79.40	35.70
**12**	34.70	-	76.10	76.40	82.30	-
**12**	36.80	-	76.10	76.30	77.40	-
**12**	37.30	-	76.10	76.30	78.20	-
**13**	26.70	26.70	40.90	37.10	41.30	34.90
**13**	26.80	26.80	40.00	36.30	41.80	30.20
**13**	28.20	28.20	41.10	40.90	41.80	34.30
**14**	28.30	-	76.40	69.50	75.10	-
**14**	31.30	-	80.50	74.90	73.20	-
**14**	29.20	-	76.30	73.20	69.30	-
**15**	15.40	15.40	29.50	25.70	26.40	17.90
**15**	16.80	16.80	36.20	30.50	29.50	22.20
**15**	13.80	13.80	31.50	29.50	26.60	21.00
**16**	31.70	31.70	76.90	89.60	69.00	44.60
**16**	33.00	33.00	77.60	75.20	71.90	40.90
**16**	32.90	32.90	84.30	73.50	76.40	39.50
**18**	27.90	27.90	76.80	74.40	37.60	32.50
**18**	26.80	26.80	76.90	77.60	37.20	33.30
**18**	28.40	28.40	77.00	77.10	45.80	36.40
**19**	28.70	-	76.40	76.10	74.90	-

CTR+						
**Subject**	**t0***	**t0****	**t2***	**t4***	**t8***	**t24****
**19**	30.30	-	76.20	77.00	72.60	-
**19**	30.10	-	76.40	76.40	68.80	-
**20**	28.80	28.80	68.40	74.20	90.30	31.20
**20**	31.10	31.10	77.10	78.00	85.20	36.90
**20**	31.30	31.30	77.10	77.40	83.40	40.00
**21**	28.00	28.00	76.60	39.90	33.80	32.30
**21**	25.00	25.00	65.20	41.30	32.40	33.60
**21**	26.00	26.00	76.70	43.20	38.10	36.00
**23**	18.60	18.60	37.60	27.90	30.30	25.50
**23**	15.50	15.50	34.00	23.40	30.30	26.00
**23**	17.80	17.80	37.90	24.50	32.70	29.60
**24**	37.10	37.10	77.10	88.30	71.10	39.40
**24**	34.70	34.70	77.10	97.40	63.20	41.70
**24**	36.60	36.60	77.00	79.20	68.50	43.60
**25**	-	38.70	-	-	-	35.60
**25**	-	39.30	-	-	-	34.90
**25**	-	40.30	-	-	-	36.90
**26**	-	30.80	-	-	-	84.00

**26**	-	28.70	-	-	-	83.20
**26**	-	32.40	-	-	-	82.20
**27**	-	19.00	-	-	-	26.30
**27**	-	17.30	-	-	-	26.50
**27**	-	18.50	-	-	-	27.80
**28**	-	23.10	-	-	-	34.10
**28**	-	27.50	-	-	-	37.60
**28**	-	26.60	-	-	-	39.80
**29**	-	29.10	-	-	-	33.70
**29**	-	25.80	-	-	-	33.10
**29**	-	24.50	-	-	-	36.10
**30**	-	35.90	-	-	-	46.20
**30**	-	35.50	-	-	-	49.30
**30**	-	38.80	-	-	-	53.80
*1st group values considered for the hydration evaluation of time-points 2h, 4h and 8h after products´application<br><br>** 2nd group: values considered for the hydration evaluation at time-point 24h after products´application						

Table S5.3 – Information regarding the hydration (A.U.) values from all the subjects at time zero (T0), and .after application of the CTR-, 2h, 4h, 8h and 24h.

CTR-					
**Subject**	**t0**	**t2**	**t4**	**t8**	**t24**
**1**	28.50	25.80	27.70	28.20	22.80
**1**	36.50	31.90	33.20	33.40	21.60
**1**	35.80	28.20	25.10	26.00	21.70
**2**	32.00	27.60	28.10	36.20	34.00
**2**	39.20	31.10	36.30	40.90	41.50
**2**	33.40	27.20	31.20	33.00	34.80
**4**	29.50	20.10	21.40	24.50	23.60
**4**	33.80	22.50	23.00	23.00	25.60
**4**	26.70	19.70	19.80	20.60	23.10
**5**	21.10	20.40	18.90	19.20	23.10
**5**	18.70	19.30	18.60	15.90	23.90
**5**	18.10	18.50	18.00	16.10	21.00
**6**	32.50	26.30	29.70	25.70	23.30
**6**	31.40	31.80	35.50	27.80	25.60
**6**	30.50	27.90	31.20	25.20	26.00
**9**	36.60	28.00	25.10	31.90	36.50
**9**	29.80	25.20	20.20	32.10	29.90
**9**	31.10	28.40	24.00	30.90	32.30
**12**	32.90	28.60	32.00	29.30	30.00
**12**	34.60	34.10	35.10	29.60	36.30

**12**	33.80	26.60	30.60	27.10	27.90
**13**	22.20	23.20	23.10	27.00	22.30
**13**	21.30	21.60	23.70	26.90	20.40
**13**	20.80	20.50	21.80	28.80	26.80
**14**	33.80	26.20	26.60	26.40	28.80
**14**	35.40	30.00	27.90	28.70	31.00
**14**	33.90	23.80	22.90	23.70	27.00
**15**	21.00	21.30	19.20	17.70	21.50
**15**	13.50	16.90	16.00	17.80	16.20
**15**	19.20	20.20	18.80	18.40	21.70
**16**	31.00	28.80	24.90	30.80	28.80
**16**	30.10	27.00	26.80	30.30	26.70
**16**	30.90	27.10	25.90	32.20	25.10
**18**	23.30	22.50	20.60	17.00	22.30
**18**	20.70	25.00	20.00	19.80	25.30
**18**	23.70	22.70	18.10	18.70	24.40

CTR-					
**Subject**	**t0**	**t2**	**t4**	**t8**	**t24**
**19**	22.10	20.30	25.00	18.00	22.90
**19**	27.20	21.20	22.10	18.30	18.20

**19**	24.60	21.80	24.40	14.00	19.10
**20**	28.20	27.70	22.80	28.40	25.60
**20**	33.30	26.50	23.20	31.10	25.60
**20**	29.50	28.40	25.70	28.60	26.00
**21**	22.40	25.60	21.40	21.60	29.60
**21**	19.90	23.70	20.10	19.00	22.20
**21**	18.00	25.60	20.10	17.00	25.60
**23**	20.00	16.00	15.50	16.30	26.30
**23**	16.50	14.00	13.20	15.40	22.90
**23**	14.70	15.20	13.70	17.50	23.40
**24**	34.30	27.00	29.80	33.50	37.90
**24**	37.00	34.30	30.70	31.90	39.30
**24**	32.30	28.30	25.70	34.20	36.70
**25**	35.10	-	-	-	35.80
**25**	39.70	-	-	-	38.00
**25**	40.50	-	-	-	38.00
**26**	37.10	-	-	-	38.40
**26**	30.30	-	-	-	35.10
**26**	32.00	-	-	-	41.60
**27**	27.10	-	-	-	29.30
**27**	24.10	-	-	-	31.90
**27**	26.50	-	-	-	32.10
**28**	22.40	-	-	-	22.60

**28**	24.20	-	-	-	23.50
**28**	25.20	-	-	-	24.40
**29**	31.40	-	-	-	29.00
**29**	30.80	-	-	-	34.70
**29**	28.00	-	-	-	33.00
**30**	25.20	-	-	-	30.70
**30**	27.00	-	-	-	29.30
**30**	30.90	-	-	-	31.10

Table S5.4 – Data analysis AADE of the hydration values from all the subjects at time zero (T0), and .after application of the product CTR-, 2h, 4h, 8h and 24h

CTR-						
**Subject**	**t0***	**t0****	**t2***	**t4***	**t8***	**t24****
**1**	28.50	-	25.80	27.70	28.20	-
**1**	36.50	-	31.90	33.20	33.40	-
**1**	35.80	-	28.20	25.10	26.00	-
**2**	32.00	32.00	27.60	28.10	36.20	34.00

**2**	39.20	39.20	31.10	36.30	40.90	41.50
**2**	33.40	33.40	27.20	31.20	33.00	34.80
**4**	29.50	-	20.10	21.40	24.50	-
**4**	33.80	-	22.50	23.00	23.00	-
**4**	26.70	-	19.70	19.80	20.60	-
**5**	21.10	21.10	20.40	18.90	19.20	23.10
**5**	18.70	18.70	19.30	18.60	15.90	23.90
**5**	18.10	18.10	18.50	18.00	16.10	21.00
**6**	32.50	-	26.30	29.70	25.70	-
**6**	31.40	-	31.80	35.50	27.80	-
**6**	30.50	-	27.90	31.20	25.20	-
**9**	36.60	36.60	28.00	25.10	31.90	36.50
**9**	29.80	29.80	25.20	20.20	32.10	29.90
**9**	31.10	31.10	28.40	24.00	30.90	32.30
**12**	32.90	-	28.60	32.00	29.30	-
**12**	34.60	-	34.10	35.10	29.60	-
**12**	33.80	-	26.60	30.60	27.10	-
**13**	22.20	22.20	23.20	23.10	27.00	22.30
**13**	21.30	21.30	21.60	23.70	26.90	20.40
**13**	20.80	20.80	20.50	21.80	28.80	26.80
**14**	33.80	-	26.20	26.60	26.40	-
**14**	35.40	-	30.00	27.90	28.70	-
**14**	33.90	-	23.80	22.90	23.70	-

**15**	21.00	21.00	21.30	19.20	17.70	21.50
**15**	13.50	13.50	16.90	16.00	17.80	16.20
**15**	19.20	19.20	20.20	18.80	18.40	21.70
**16**	31.00	31.00	28.80	24.90	30.80	28.80
**16**	30.10	30.10	27.00	26.80	30.30	26.70
**16**	30.90	30.90	27.10	25.90	32.20	25.10
**18**	23.30	23.30	22.50	20.60	17.00	22.30
**18**	20.70	20.70	25.00	20.00	19.80	25.30
**18**	23.70	23.70	22.70	18.10	18.70	24.40

CTR-						
**Subject**	**t0***	**t0****	**t2***	**t4***	**t8***	**t24****
**19**	22.10	-	20.30	25.00	18.00	-
**19**	27.20	-	21.20	22.10	18.30	-
**19**	24.60	-	21.80	24.40	14.00	-
**20**	28.20	28.20	27.70	22.80	28.40	25.60
**20**	33.30	33.30	26.50	23.20	31.10	25.60
**20**	29.50	29.50	28.40	25.70	28.60	26.00
**21**	22.40	22.40	25.60	21.40	21.60	29.60
**21**	19.90	19.90	23.70	20.10	19.00	22.20

**21**	18.00	18.00	25.60	20.10	17.00	25.60
**23**	20.00	20.00	16.00	15.50	16.30	26.30
**23**	16.50	16.50	14.00	13.20	15.40	22.90
**23**	14.70	14.70	15.20	13.70	17.50	23.40
**24**	34.30	34.30	27.00	29.80	33.50	37.90
**24**	37.00	37.00	34.30	30.70	31.90	39.30
**24**	32.30	32.30	28.30	25.70	34.20	36.70
**25**	-	35.10	-	-	-	35.80
**25**	-	39.70	-	-	-	38.00
**25**	-	40.50	-	-	-	38.00
**26**	-	37.10	-	-	-	38.40
**26**	-	30.30	-	-	-	35.10
**26**	-	32.00	-	-	-	41.60
**27**	-	27.10	-	-	-	29.30
**27**	-	24.10	-	-	-	31.90
**27**	-	26.50	-	-	-	32.10
**28**	-	22.40	-	-	-	22.60
**28**	-	24.20	-	-	-	23.50
**28**	-	25.20	-	-	-	24.40
**29**	-	31.40	-	-	-	29.00
**29**	-	30.80	-	-	-	34.70
**29**	-	28.00	-	-	-	33.00
**30**	-	25.20	-	-	-	30.70

**30**	-	27.00	-	-	-	29.30
**30**	-	30.90	-	-	-	31.10

*1st group: values considered for the hydration evaluation at time-points 2 hours, 4 hours and 8 hours after products' application

**2nd group: values considered for the hydration evaluation at time-point 24 hours after products' application

Table S5.5 – Information regarding the hydration (A.U.) values from all the subjects at time zero (T0), and .after application of the F4, 2h, 4h, 8h and 24h.

F4					
**Subject**	**t0**	**t2**	**t4**	**t8**	**t24**
**1**	34.60	37.40	34.10	27.50	21.00
**1**	34.10	34.20	35.70	26.30	24.00
**1**	27.90	27.60	29.70	21.30	19.70
**2**	47.10	39.80	43.10	39.70	43.30
**2**	47.30	37.20	42.10	44.30	42.40
**2**	56.10	42.10	42.60	41.20	48.00
**4**	34.40	28.60	32.00	25.60	25.50
**4**	28.50	31.70	30.50	24.90	21.00
**4**	28.60	23.00	31.90	27.70	26.00

**5**	17.10	19.60	20.90	17.30	17.60
**5**	20.60	21.80	22.70	12.90	19.50
**5**	16.20	20.40	21.00	15.20	18.90
**6**	26.80	30.80	29.70	24.70	25.10
**6**	27.90	32.00	31.30	24.40	24.50
**6**	28.40	33.60	31.40	22.60	24.70
**9**	31.40	25.20	22.80	27.00	32.30
**9**	32.20	25.00	21.70	27.70	26.50
**9**	30.00	27.10	24.20	26.60	29.80
**12**	34.90	23.00	28.10	26.20	29.30
**12**	34.80	25.70	29.50	24.80	22.90
**12**	36.30	27.70	35.00	21.40	26.70
**13**	23.40	24.50	23.70	28.90	27.10
**13**	22.10	26.50	26.90	26.60	26.50
**13**	22.20	23.20	22.90	27.60	24.00
**14**	39.30	27.10	36.20	35.30	30.70
**14**	31.70	29.90	30.20	27.20	27.90
**14**	35.10	30.20	32.60	29.70	28.60
**15**	16.10	21.30	19.50	16.00	20.80
**15**	16.10	17.10	14.70	16.40	17.00
**15**	18.90	20.40	18.30	17.00	19.50
**16**	24.20	20.80	16.80	23.50	20.10
**16**	25.00	15.30	14.50	22.40	19.00

**16**	23.80	13.70	17.50	22.70	20.00
**18**	25.20	37.30	37.80	31.70	27.30
**18**	26.80	34.20	30.50	27.10	24.50
**18**	25.50	34.80	32.10	29.20	26.30
**19**	28.10	22.60	24.90	18.00	23.60

F4					
**Subject**	**t0**	**t2**	**t4**	**t8**	**t24**
**19**	25.90	20.80	23.70	22.50	21.70
**19**	22.90	21.00	21.90	16.90	18.30
**20**	33.70	32.00	30.30	36.00	39.20
**20**	32.80	32.10	31.00	39.50	36.50
**20**	31.70	26.10	31.10	33.10	33.00
**21**	23.80	18.90	18.50	20.00	24.30
**21**	25.30	24.70	25.60	22.60	27.20
**21**	27.50	24.10	20.90	24.50	25.70
**23**	15.30	14.20	13.30	21.00	17.20
**23**	18.50	13.70	11.30	20.50	19.10
**23**	16.60	16.10	11.80	20.00	16.40
**24**	35.60	32.60	33.40	38.60	32.90
**24**	33.20	32.50	28.30	37.20	33.80
**24**	34.10	34.10	35.80	39.60	36.80

**25**	36.10	-	-	-	29.50
**25**	33.20	-	-	-	26.60
**25**	39.00	-	-	-	32.40
**26**	35.70	-	-	-	41.60
**26**	28.80	-	-	-	41.70
**26**	33.80	-	-	-	49.70
**27**	28.80	-	-	-	37.40
**27**	27.80	-	-	-	38.30
**27**	35.30	-	-	-	31.30
**28**	28.40	-	-	-	30.50
**28**	27.70	-	-	-	31.80
**28**	34.60	-	-	-	31.60
**29**	30.30	-	-	-	29.70
**29**	26.50	-	-	-	34.00
**29**	27.00	-	-	-	32.30
**30**	24.60	-	-	-	26.70
**30**	27.30	-	-	-	26.00
**30**	26.90	-	-	-	25.40

Table S5.6 – Data analysis AADE of the hydration values from all the subjects at time zero (T0), and .after application of the F4, 2h, 4h, 8h and 24h.

F4						
Subject	t0*	t0**	t2*	t4*	t8*	t24**
1	34.60	-	37.40	34.10	27.50	-
1	34.10	-	34.20	35.70	26.30	-
1	27.90	-	27.60	29.70	21.30	-
2	47.10	47.10	39.80	43.10	39.70	43.30
2	47.30	47.30	37.20	42.10	44.30	42.40
2	56.10	56.10	42.10	42.60	41.20	48.00
4	34.40	-	28.60	32.00	25.60	-
4	28.50	-	31.70	30.50	24.90	-
4	28.60	-	23.00	31.90	27.70	-
5	17.10	17.10	19.60	20.90	17.30	17.60
5	20.60	20.60	21.80	22.70	12.90	19.50
5	16.20	16.20	20.40	21.00	15.20	18.90
6	26.80	-	30.80	29.70	24.70	-
6	27.90	-	32.00	31.30	24.40	-
6	28.40	-	33.60	31.40	22.60	-
9	31.40	31.40	25.20	22.80	27.00	32.30
9	32.20	32.20	25.00	21.70	27.70	26.50
9	30.00	30.00	27.10	24.20	26.60	29.80

**12**	34.90	-	23.00	28.10	26.20	-
**12**	34.80	-	25.70	29.50	24.80	-
**12**	36.30	-	27.70	35.00	21.40	-
**13**	23.40	23.40	24.50	23.70	28.90	27.10
**13**	22.10	22.10	26.50	26.90	26.60	26.50
**13**	22.20	22.20	23.20	22.90	27.60	24.00
**14**	39.30	-	27.10	36.20	35.30	-
**14**	31.70	-	29.90	30.20	27.20	-
**14**	35.10	-	30.20	32.60	29.70	-
**15**	16.10	16.10	21.30	19.50	16.00	20.80
**15**	16.10	16.10	17.10	14.70	16.40	17.00
**15**	18.90	18.90	20.40	18.30	17.00	19.50
**16**	24.20	24.20	20.80	16.80	23.50	20.10
**16**	25.00	25.00	15.30	14.50	22.40	19.00
**16**	23.80	23.80	13.70	17.50	22.70	20.00
**18**	25.20	25.20	37.30	37.80	31.70	27.30
**18**	26.80	26.80	34.20	30.50	27.10	24.50
**18**	25.50	25.50	34.80	32.10	29.20	26.30
**19**	28.10	-	22.60	24.90	18.00	-

**F4**						
**Subject**	**t0***	**t0****	**t2***	**t4***	**t8***	**t24****

**19**	25.90	-	20.80	23.70	22.50	-
**19**	22.90	-	21.00	21.90	16.90	-
**20**	33.70	33.70	32.00	30.30	36.00	39.20
**20**	32.80	32.80	32.10	31.00	39.50	36.50
**20**	31.70	31.70	26.10	31.10	33.10	33.00
**21**	23.80	23.80	18.90	18.50	20.00	24.30
**21**	25.30	25.30	24.70	25.60	22.60	27.20
**21**	27.50	27.50	24.10	20.90	24.50	25.70
**23**	15.30	15.30	14.20	13.30	21.00	17.20
**23**	18.50	18.50	13.70	11.30	20.50	19.10
**23**	16.60	16.60	16.10	11.80	20.00	16.40
**24**	35.60	35.60	32.60	33.40	38.60	32.90
**24**	33.20	33.20	32.50	28.30	37.20	33.80
**24**	34.10	34.10	34.10	35.80	39.60	36.80
**25**	-	36.10	-	-	-	29.50
**25**	-	33.20	-	-	-	26.60
**25**	-	39.00	-	-	-	32.40
**26**	-	35.70	-	-	-	41.60
**26**	-	28.80	-	-	-	41.70
**26**	-	33.80	-	-	-	49.70
**27**	-	28.80	-	-	-	37.40
**27**	-	27.80	-	-	-	38.30
**27**	-	35.30	-	-	-	31.30

**28**	-	28.40	-	-	-	30.50
**28**	-	27.70	-	-	-	31.80
**28**	-	34.60	-	-	-	31.60
**29**	-	30.30	-	-	-	29.70
**29**	-	26.50	-	-	-	34.00
**29**	-	27.00	-	-	-	32.30
**30**	-	24.60	-	-	-	26.70
**30**	-	27.30	-	-	-	26.00
**30**	-	26.90	-	-	-	25.40

*1st group: values considered for the hydration evaluation at time-points 2 hours, 4 hours and 8 hours after products' application

**2nd group: values considered for the hydration evaluation at time-point 24 hours after products' application

Table S5.7 – Information regarding the hydration (A.U.) values from all the subjects at time zero (T0), and .after application of the F1, 2h, 4h, 8h and 24h.

**F1**					
**Subject**	**t0**	**t2**	**t4**	**t8**	**t24**
**1**	40.60	31.30	32.30	29.90	28.00

**1**	37.20	25.70	24.70	24.90	21.30
**1**	39.50	31.00	31.50	29.10	28.20
**2**	38.10	24.40	28.80	33.00	34.20
**2**	32.40	24.40	25.90	31.10	29.90
**2**	36.80	23.70	27.10	32.60	32.70
**4**	38.30	36.30	35.00	37.60	32.40
**4**	44.30	33.30	37.10	34.20	30.90
**4**	37.80	32.40	34.00	35.50	32.60
**5**	23.10	28.50	27.40	24.50	23.80
**5**	19.20	27.50	24.20	18.90	18.00
**5**	20.60	25.70	21.40	18.10	20.00
**6**	28.90	35.40	39.20	25.40	24.40
**6**	25.80	30.10	38.10	27.40	24.30
**6**	27.50	32.70	38.00	24.70	27.40
**9**	33.60	32.70	28.90	32.90	34.90
**9**	29.90	28.50	21.40	27.80	25.80
**9**	32.10	31.10	29.70	35.70	32.90
**12**	31.80	37.30	36.30	25.80	30.60
**12**	33.60	28.90	29.10	26.70	27.60
**12**	28.10	34.80	33.30	22.50	28.90
**13**	20.50	20.60	24.50	22.70	20.20
**13**	22.90	21.70	23.30	28.40	24.20
**13**	23.90	20.20	20.30	28.70	22.90
**14**	36.40	26.80	30.70	25.70	20.50
**14**	28.90	25.30	26.00	23.40	18.50
**14**	29.30	26.60	28.80	25.40	19.10
**15**	17.70	19.80	15.40	20.90	22.20

**15**	16.40	17.30	12.70	13.60	13.70
**15**	19.50	13.70	14.50	22.80	18.00
**16**	30.50	31.80	30.40	33.00	26.20
**16**	35.20	31.10	29.30	31.80	25.00
**16**	30.90	30.50	32.50	35.40	23.50
**18**	28.60	24.90	29.50	27.80	29.90
**18**	29.10	28.70	27.20	29.70	30.60
**18**	27.80	27.00	29.20	27.30	32.10
**19**	28.50	24.70	21.20	18.70	20.20
**19**	27.10	24.80	17.20	17.40	23.70
**19**	25.10	20.40	23.10	11.90	21.40
**20**	32.90	26.60	21.60	29.30	26.90

**F1**					
**Subject**	**t0**	**t2**	**t4**	**t8**	**t24**
**20**	30.70	25.60	22.30	29.60	28.90
**20**	32.00	29.10	22.90	25.50	34.90
**21**	28.80	27.60	27.70	28.40	30.40
**21**	25.30	32.00	31.80	28.60	28.20
**21**	25.60	27.30	24.80	27.50	25.20
**23**	17.80	14.20	14.20	19.00	18.00
**23**	15.70	17.00	13.60	17.90	17.00
**23**	18.60	16.40	14.00	18.40	16.70

**24**	23.50	28.40	21.40	29.80	23.70
**24**	25.50	25.70	24.00	31.70	26.00
**24**	28.40	27.60	27.00	35.60	29.90
**25**	27.80	-	-	-	24.90
**25**	31.70	-	-	-	26.30
**25**	33.00	-	-	-	26.30
**26**	29.00	-	-	-	40.70
**26**	34.00	-	-	-	42.60
**26**	27.50	-	-	-	37.30
**27**	22.80	-	-	-	23.40
**27**	21.10	-	-	-	24.90
**27**	23.70	-	-	-	27.40
**28**	21.60	-	-	-	21.40
**28**	23.00	-	-	-	21.50
**28**	25.40	-	-	-	22.00
**29**	39.30	-	-	-	41.70
**29**	41.70	-	-	-	42.60
**29**	42.10	-	-	-	43.00
**30**	32.30	-	-	-	33.30
**30**	29.80	-	-	-	31.70
**30**	33.20	-	-	-	33.60

Table S5.8 – Data analysis AADE of the hydration values from all the subjects at time zero (T0), and .after application of the F1, 2h, 4h, 8h and 24h.

F1						
**Subject**	**t0***	**t0****	**t2***	**t4***	**t8***	**t24****
**1**	40.60	-	31.30	32.30	29.90	-
**1**	37.20	-	25.70	24.70	24.90	-
**1**	39.50	-	31.00	31.50	29.10	-
**2**	38.10	38.10	24.40	28.80	33.00	34.20
**2**	32.40	32.40	24.40	25.90	31.10	29.90
**2**	36.80	36.80	23.70	27.10	32.60	32.70
**4**	38.30	-	36.30	35.00	37.60	-
**4**	44.30	-	33.30	37.10	34.20	-
**4**	37.80	-	32.40	34.00	35.50	-
**5**	23.10	23.10	28.50	27.40	24.50	23.80
**5**	19.20	19.20	27.50	24.20	18.90	18.00
**5**	20.60	20.60	25.70	21.40	18.10	20.00
**6**	28.90	-	35.40	39.20	25.40	-
**6**	25.80	-	30.10	38.10	27.40	-
**6**	27.50	-	32.70	38.00	24.70	-
**9**	33.60	33.60	32.70	28.90	32.90	34.90
**9**	29.90	29.90	28.50	21.40	27.80	25.80
**9**	32.10	32.10	31.10	29.70	35.70	32.90
**12**	31.80	-	37.30	36.30	25.80	-
**12**	33.60	-	28.90	29.10	26.70	-

**12**	28.10	-	34.80	33.30	22.50	-
**13**	20.50	20.50	20.60	24.50	22.70	20.20
**13**	22.90	22.90	21.70	23.30	28.40	24.20
**13**	23.90	23.90	20.20	20.30	28.70	22.90
**14**	36.40	-	26.80	30.70	25.70	-
**14**	28.90	-	25.30	26.00	23.40	-
**14**	29.30	-	26.60	28.80	25.40	-
**15**	17.70	17.70	19.80	15.40	20.90	22.20
**15**	16.40	16.40	17.30	12.70	13.60	13.70
**15**	19.50	19.50	13.70	14.50	22.80	18.00
**16**	30.50	30.50	31.80	30.40	33.00	26.20
**16**	35.20	35.20	31.10	29.30	31.80	25.00
**16**	30.90	30.90	30.50	32.50	35.40	23.50
**18**	28.60	28.60	24.90	29.50	27.80	29.90
**18**	29.10	29.10	28.70	27.20	29.70	30.60
**18**	27.80	27.80	27.00	29.20	27.30	32.10
**19**	28.50	-	24.70	21.20	18.70	-

F1						
**Subject**	**t0***	**t0****	**t2***	**t4***	**t8***	**t24****
**19**	27.10	-	24.80	17.20	17.40	-
**19**	25.10	-	20.40	23.10	11.90	-

**20**	32.90	32.90	26.60	21.60	29.30	26.90
**20**	30.70	30.70	25.60	22.30	29.60	28.90
**20**	32.00	32.00	29.10	22.90	25.50	34.90
**21**	28.80	28.80	27.60	27.70	28.40	30.40
**21**	25.30	25.30	32.00	31.80	28.60	28.20
**21**	25.60	25.60	27.30	24.80	27.50	25.20
**23**	17.80	17.80	14.20	14.20	19.00	18.00
**23**	15.70	15.70	17.00	13.60	17.90	17.00
**23**	18.60	18.60	16.40	14.00	18.40	16.70
**24**	23.50	23.50	28.40	21.40	29.80	23.70
**24**	25.50	25.50	25.70	24.00	31.70	26.00
**24**	28.40	28.40	27.60	27.00	35.60	29.90
**25**	-	27.80	-	-	-	24.90
**25**	-	31.70	-	-	-	26.30
**25**	-	33.00	-	-	-	26.30
**26**	-	29.00	-	-	-	40.70
**26**	-	34.00	-	-	-	42.60
**26**	-	27.50	-	-	-	37.30
**27**	-	22.80	-	-	-	23.40
**27**	-	21.10	-	-	-	24.90
**27**	-	23.70	-	-	-	27.40
**28**	-	21.60	-	-	-	21.40
**28**	-	23.00	-	-	-	21.50

**28**	-	25.40	-	-	-	22.00
**29**	-	39.30	-	-	-	41.70
**29**	-	41.70	-	-	-	42.60
**29**	-	42.10	-	-	-	43.00
**30**	-	32.30	-	-	-	33.30
**30**	-	29.80	-	-	-	31.70
**30**	-	33.20	-	-	-	33.60

*1st group: values considered for the hydration evaluation at time-points 2 hours, 4 hours and 8 hours after products' application

**2nd group: values considered for the hydration evaluation at time-point 24 hours after products' application

Table S5.9 – Information regarding the hydration (A.U.) values from all the subjects at time zero (T0), and .after application of the F2, 2h, 4h, 8h and 24h.

**F2**					
**Subject**	**t0**	**t2**	**t4**	**t8**	**t24**
**1**	34.60	33.80	32.30	31.50	28.90
**1**	34.20	26.20	28.40	25.90	25.80
**1**	37.10	29.20	31.10	31.60	26.70
**2**	43.60	44.30	41.70	42.10	40.50
**2**	43.60	44.70	35.90	41.80	32.00

**2**	41.80	33.50	41.50	50.60	34.40
**4**	31.00	29.10	29.40	32.70	27.60
**4**	30.80	25.20	27.80	31.80	25.00
**4**	31.40	30.80	30.00	33.60	29.90
**5**	25.60	25.40	22.00	15.90	25.20
**5**	21.50	23.40	15.50	16.90	19.20
**5**	21.30	21.90	13.90	12.30	17.60
**6**	28.80	30.60	28.60	23.10	25.10
**6**	28.60	34.10	32.10	25.10	27.00
**6**	26.40	31.00	30.10	25.30	27.10
**9**	35.60	31.60	23.70	32.10	35.00
**9**	34.70	33.60	26.00	33.00	32.40
**9**	32.50	33.00	24.20	35.40	30.10
**12**	36.40	36.60	31.10	26.50	23.40
**12**	36.20	37.70	35.80	25.90	26.20
**12**	31.70	33.50	32.90	30.70	22.50
**13**	18.00	22.60	21.80	25.30	21.40
**13**	18.80	19.20	15.20	19.70	15.90
**13**	19.00	24.70	22.70	23.80	24.00
**14**	34.60	32.20	32.40	32.30	28.20
**14**	34.20	32.80	36.50	35.00	28.30
**14**	36.90	34.40	35.60	35.00	31.10
**15**	15.80	13.90	17.80	13.70	15.90

**15**	14.50	17.10	18.60	17.20	21.00
**15**	15.10	14.30	12.80	13.90	14.60
**16**	25.70	22.60	23.90	30.40	27.20
**16**	25.40	28.50	26.60	29.60	22.20
**16**	23.40	22.30	23.30	30.80	20.50
**18**	24.00	14.00	15.50	15.00	19.70
**18**	17.90	13.90	15.80	17.00	17.20
**18**	24.10	14.50	14.30	16.40	21.40
**19**	31.10	31.10	32.20	26.30	25.00

F2					
**Subject**	**t0**	**t2**	**t4**	**t8**	**t24**
**19**	28.40	27.10	28.00	22.60	25.20
**19**	28.20	26.20	27.50	18.40	24.00
**20**	31.20	28.30	29.60	24.00	29.90
**20**	29.10	27.50	26.20	33.40	27.10
**20**	31.90	31.70	32.10	32.00	28.40
**21**	27.30	27.00	25.00	22.80	24.70
**21**	25.70	29.70	23.40	21.60	30.80
**21**	24.60	24.00	22.00	23.50	26.70
**23**	20.10	18.90	12.90	19.10	21.80
**23**	19.30	16.40	15.00	18.20	18.80

**23**	17.20	18.50	14.50	20.30	21.70
**24**	32.20	23.70	28.50	30.20	30.60
**24**	29.80	26.40	27.10	29.30	29.30
**24**	30.20	22.80	25.60	28.40	30.40
**25**	35.90	-	-	-	28.00
**25**	36.20	-	-	-	32.00
**25**	37.70	-	-	-	31.50
**26**	34.70	-	-	-	43.80
**26**	29.60	-	-	-	35.70
**26**	28.00	-	-	-	40.30
**27**	28.80	-	-	-	27.90
**27**	22.90	-	-	-	29.80
**27**	26.70	-	-	-	33.30
**28**	23.70	-	-	-	25.10
**28**	22.90	-	-	-	25.20
**28**	28.60	-	-	-	23.90
**29**	35.40	-	-	-	34.70
**29**	30.30	-	-	-	32.90
**29**	31.40	-	-	-	39.00
**30**	31.80	-	-	-	28.20
**30**	24.40	-	-	-	24.00
**30**	29.20	-	-	-	27.20

Table S5.10 – Data analysis AADE of the hydration values from all the subjects at time zero (T0), and .after application of the F2, 2h, 4h, 8h and 24h.

F2						
**Subject**	**t0***	**t0****	**t2***	**t4***	**t8***	**t24****
**1**	34.60	-	33.80	32.30	31.50	-
**1**	34.20	-	26.20	28.40	25.90	-
**1**	37.10	-	29.20	31.10	31.60	-
**2**	43.60	43.60	44.30	41.70	42.10	40.50
**2**	43.60	43.60	44.70	35.90	41.80	32.00
**2**	41.80	41.80	33.50	41.50	50.60	34.40
**4**	31.00	-	29.10	29.40	32.70	-
**4**	30.80	-	25.20	27.80	31.80	-
**4**	31.40	-	30.80	30.00	33.60	-
**5**	25.60	25.60	25.40	22.00	15.90	25.20
**5**	21.50	21.50	23.40	15.50	16.90	19.20
**5**	21.30	21.30	21.90	13.90	12.30	17.60
**6**	28.80	-	30.60	28.60	23.10	-

**6**	28.60	-	34.10	32.10	25.10	-
**6**	26.40	-	31.00	30.10	25.30	-
**9**	35.60	35.60	31.60	23.70	32.10	35.00
**9**	34.70	34.70	33.60	26.00	33.00	32.40
**9**	32.50	32.50	33.00	24.20	35.40	30.10
**12**	36.40	-	36.60	31.10	26.50	-
**12**	36.20	-	37.70	35.80	25.90	-
**12**	31.70	-	33.50	32.90	30.70	-
**13**	18.00	18.00	22.60	21.80	25.30	21.40
**13**	18.80	18.80	19.20	15.20	19.70	15.90
**13**	19.00	19.00	24.70	22.70	23.80	24.00
**14**	34.60	-	32.20	32.40	32.30	-
**14**	34.20	-	32.80	36.50	35.00	-
**14**	36.90	-	34.40	35.60	35.00	-
**15**	15.80	15.80	13.90	17.80	13.70	15.90
**15**	14.50	14.50	17.10	18.60	17.20	21.00
**15**	15.10	15.10	14.30	12.80	13.90	14.60
**16**	25.70	25.70	22.60	23.90	30.40	27.20
**16**	25.40	25.40	28.50	26.60	29.60	22.20
**16**	23.40	23.40	22.30	23.30	30.80	20.50
**18**	24.00	24.00	14.00	15.50	15.00	19.70
**18**	17.90	17.90	13.90	15.80	17.00	17.20
**18**	24.10	24.10	14.50	14.30	16.40	21.40
**19**	31.10	-	31.10	32.20	26.30	-

F2						
**Subject**	**t0***	**t0****	**t2***	**t4***	**t8***	**t24****
**19**	28.40	-	27.10	28.00	22.60	-
**19**	28.20	-	26.20	27.50	18.40	-
**20**	31.20	31.20	28.30	29.60	24.00	29.90
**20**	29.10	29.10	27.50	26.20	33.40	27.10
**20**	31.90	31.90	31.70	32.10	32.00	28.40
**21**	27.30	27.30	27.00	25.00	22.80	24.70
**21**	25.70	25.70	29.70	23.40	21.60	30.80
**21**	24.60	24.60	24.00	22.00	23.50	26.70
**23**	20.10	20.10	18.90	12.90	19.10	21.80
**23**	19.30	19.30	16.40	15.00	18.20	18.80
**23**	17.20	17.20	18.50	14.50	20.30	21.70
**24**	32.20	32.20	23.70	28.50	30.20	30.60
**24**	29.80	29.80	26.40	27.10	29.30	29.30
**24**	30.20	30.20	22.80	25.60	28.40	30.40
**25**	-	35.90	-	-	-	28.00
**25**	-	36.20	-	-	-	32.00
**25**	-	37.70	-	-	-	31.50
**26**	-	34.70	-	-	-	43.80
**26**	-	29.60	-	-	-	35.70

**26**	-	28.00	-	-	-	40.30
**27**	-	28.80	-	-	-	27.90
**27**	-	22.90	-	-	-	29.80
**27**	-	26.70	-	-	-	33.30
**28**	-	23.70	-	-	-	25.10
**28**	-	22.90	-	-	-	25.20
**28**	-	28.60	-	-	-	23.90
**29**	-	35.40	-	-	-	34.70
**29**	-	30.30	-	-	-	32.90
**29**	-	31.40	-	-	-	39.00
**30**	-	31.80	-	-	-	28.20
**30**	-	24.40	-	-	-	24.00
**30**	-	29.20	-	-	-	27.20
*1st group: values considered for the hydration evaluation at time-points 2 hours, 4 hours and 8 hours after products' application<br><br>**2nd group: values considered for the hydration evaluation at time-point 24 hours after products' application						

Table S5.11 – Information regarding the hydration (A.U.) values from all the subjects at time zero (T0), and .after application of the F3, 2h, 4h, 8h and 24h.

F3					
**Subject**	**t0**	**t2**	**t4**	**t8**	**t24**
**1**	35.50	27.60	35.00	30.80	26.10
**1**	33.00	23.70	34.60	29.80	25.20
**1**	39.60	29.60	33.40	29.30	24.40
**2**	25.70	33.50	32.30	29.50	28.20
**2**	34.70	33.80	34.20	38.00	32.20
**2**	26.50	33.80	29.40	35.50	26.20
**4**	36.00	32.90	34.80	33.70	32.10
**4**	36.00	28.80	32.60	31.70	30.00
**4**	33.50	30.80	30.60	33.70	28.50
**5**	17.70	24.20	23.40	24.10	27.30
**5**	20.80	27.00	27.90	19.00	25.60
**5**	16.90	25.80	23.40	23.10	27.00
**6**	28.50	35.00	32.80	24.10	23.20
**6**	23.80	27.10	28.40	23.80	23.20
**6**	30.90	31.80	32.50	27.20	28.60
**9**	37.60	28.40	21.40	27.80	30.30
**9**	36.60	24.40	19.50	32.60	28.20
**9**	35.30	28.10	24.50	29.50	28.00
**12**	41.90	36.30	34.90	28.70	29.40
**12**	33.50	30.30	32.30	29.70	26.70

**12**	36.10	36.10	37.30	27.70	30.30
**13**	24.20	28.80	25.60	26.60	26.20
**13**	26.80	32.90	32.40	31.70	25.60
**13**	20.80	29.90	23.30	27.20	31.00
**14**	35.00	31.60	30.50	30.10	30.60
**14**	40.50	32.50	34.40	33.20	33.40
**14**	40.50	30.30	28.90	24.70	28.50
**15**	18.40	16.90	17.00	18.80	21.20
**15**	14.40	13.70	15.00	14.20	16.20
**15**	17.70	16.50	17.80	17.90	21.60
**16**	35.30	37.40	37.30	39.50	30.80
**16**	30.00	42.40	31.70	35.00	30.20
**16**	31.60	35.80	35.00	44.00	30.30
**18**	24.40	19.60	20.60	19.10	21.00
**18**	24.60	23.80	22.50	23.70	21.80
**18**	27.70	27.90	24.20	25.90	25.00
**19**	25.40	21.00	24.50	14.50	22.50

F3					
**Subject**	**t0**	**t2**	**t4**	**t8**	**t24**
**19**	28.90	17.30	20.90	18.00	19.70

**19**	30.40	18.80	20.90	17.20	19.50
**20**	24.20	21.50	20.00	33.30	19.30
**20**	27.10	17.00	14.40	27.30	29.30
**20**	29.50	21.00	16.50	33.70	21.10
**21**	31.30	32.10	29.90	31.20	28.70
**21**	26.30	30.20	25.20	29.10	24.50
**21**	23.80	24.10	28.00	30.00	26.90
**23**	15.80	16.20	10.50	12.70	23.60
**23**	19.20	16.50	12.40	13.50	19.00
**23**	15.10	15.60	13.20	16.70	20.30
**24**	29.50	35.90	29.40	37.00	27.90
**24**	31.20	38.10	29.30	29.70	33.50
**24**	33.90	36.40	30.70	33.60	31.10
**25**	34.50	-	-	-	36.70
**25**	41.10	-	-	-	35.70
**25**	34.70	-	-	-	34.90
**26**	34.40	-	-	-	41.30
**26**	29.10	-	-	-	36.60
**26**	30.30	-	-	-	38.40
**27**	28.70	-	-	-	30.90
**27**	28.90	-	-	-	29.80
**27**	26.00	-	-	-	31.10
**28**	24.40	-	-	-	26.70

**28**	27.80	-	-	-	23.70
**28**	27.50	-	-	-	26.80
**29**	38.80	-	-	-	35.40
**29**	35.70	-	-	-	34.70
**29**	42.10	-	-	-	39.40
**30**	33.20	-	-	-	28.90
**30**	35.00	-	-	-	33.70
**30**	35.70	-	-	-	33.30

Table S5.12 – Data analysis AADE of the hydration values from all the subjects at time zero (T0), and .after application of the F3, 2h, 4h, 8h and 24h

F3						
**Subject**	**t0***	**t0****	**t2***	**t4***	**t8***	**t24****
**1**	35.50	-	27.60	35.00	30.80	-
**1**	33.00	-	23.70	34.60	29.80	-
**1**	39.60	-	29.60	33.40	29.30	-
**2**	25.70	25.70	33.50	32.30	29.50	28.20

**2**	34.70	34.70	33.80	34.20	38.00	32.20
**2**	26.50	26.50	33.80	29.40	35.50	26.20
**4**	36.00	-	32.90	34.80	33.70	-
**4**	36.00	-	28.80	32.60	31.70	-
**4**	33.50	-	30.80	30.60	33.70	-
**5**	17.70	17.70	24.20	23.40	24.10	27.30
**5**	20.80	20.80	27.00	27.90	19.00	25.60
**5**	16.90	16.90	25.80	23.40	23.10	27.00
**6**	28.50	-	35.00	32.80	24.10	-
**6**	23.80	-	27.10	28.40	23.80	-
**6**	30.90	-	31.80	32.50	27.20	-
**9**	37.60	37.60	28.40	21.40	27.80	30.30
**9**	36.60	36.60	24.40	19.50	32.60	28.20
**9**	35.30	35.30	28.10	24.50	29.50	28.00
**12**	41.90	-	36.30	34.90	28.70	-
**12**	33.50	-	30.30	32.30	29.70	-
**12**	36.10	-	36.10	37.30	27.70	-
**13**	24.20	24.20	28.80	25.60	26.60	26.20
**13**	26.80	26.80	32.90	32.40	31.70	25.60
**13**	20.80	20.80	29.90	23.30	27.20	31.00
**14**	35.00	-	31.60	30.50	30.10	-
**14**	40.50	-	32.50	34.40	33.20	-
**14**	40.50	-	30.30	28.90	24.70	-

**15**	18.40	18.40	16.90	17.00	18.80	21.20
**15**	14.40	14.40	13.70	15.00	14.20	16.20
**15**	17.70	17.70	16.50	17.80	17.90	21.60
**16**	35.30	35.30	37.40	37.30	39.50	30.80
**16**	30.00	30.00	42.40	31.70	35.00	30.20
**16**	31.60	31.60	35.80	35.00	44.00	30.30
**18**	24.40	24.40	19.60	20.60	19.10	21.00
**18**	24.60	24.60	23.80	22.50	23.70	21.80
**18**	27.70	27.70	27.90	24.20	25.90	25.00
**19**	25.40	-	21.00	24.50	14.50	-

F3						
Subject	t0*	t0**	t2*	t4*	t8*	t24**
**19**	28.90	-	17.30	20.90	18.00	-
**19**	30.40	-	18.80	20.90	17.20	-
**20**	24.20	24.20	21.50	20.00	33.30	19.30
**20**	27.10	27.10	17.00	14.40	27.30	29.30
**20**	29.50	29.50	21.00	16.50	33.70	21.10
**21**	31.30	31.30	32.10	29.90	31.20	28.70
**21**	26.30	26.30	30.20	25.20	29.10	24.50
**21**	23.80	23.80	24.10	28.00	30.00	26.90
**23**	15.80	15.80	16.20	10.50	12.70	23.60

**23**	19.20	19.20	16.50	12.40	13.50	19.00
**23**	15.10	15.10	15.60	13.20	16.70	20.30
**24**	29.50	29.50	35.90	29.40	37.00	27.90
**24**	31.20	31.20	38.10	29.30	29.70	33.50
**24**	33.90	33.90	36.40	30.70	33.60	31.10
**25**	-	34.50	-	-	-	36.70
**25**	-	41.10	-	-	-	35.70
**25**	-	34.70	-	-	-	34.90
**26**	-	34.40	-	-	-	41.30
**26**	-	29.10	-	-	-	36.60
**26**	-	30.30	-	-	-	38.40
**27**	-	28.70	-	-	-	30.90
**27**	-	28.90	-	-	-	29.80
**27**	-	26.00	-	-	-	31.10
**28**	-	24.40	-	-	-	26.70
**28**	-	27.80	-	-	-	23.70
**28**	-	27.50	-	-	-	26.80
**29**	-	38.80	-	-	-	35.40
**29**	-	35.70	-	-	-	34.70
**29**	-	42.10	-	-	-	39.40
**30**	-	33.20	-	-	-	28.90
**30**	-	35.00	-	-	-	33.70
**30**	-	35.70	-	-	-	33.30

*1st group: values considered for the hydration evaluation at time-points 2 hours, 4 hours and 8 hours after products' application

**2nd group: values considered for the hydration evaluation at time-point 24 hours after products' application
